# Exploring the Electronic and Mechanical Properties of TPDH Nanotube: Insights from Ab Initio and Classical Molecular Dynamics Simulations

**DOI:** 10.1021/acsomega.4c05614

**Published:** 2024-12-11

**Authors:** Juan Gomez Quispe, Douglas Soares Galvao, Pedro Alves da Silva Autreto

**Affiliations:** † Electronic Structure and Atomistic Dynamics Interdisciplinary Group (GEEDAI), Center for Natural and Human Sciences (CCNH), Federal University of ABC (UFABC), Avenida dos Estados 5001, 09210-580 Santo Andre, Sao Paulo, Brazil; ‡ Applied Physics Department and Center for Computing in Engineering and Sciences, State University of Campinas, Rua Sergio Buarque de Holanda 777, 13083-859 Campinas, Sao Paulo, Brazil

## Abstract

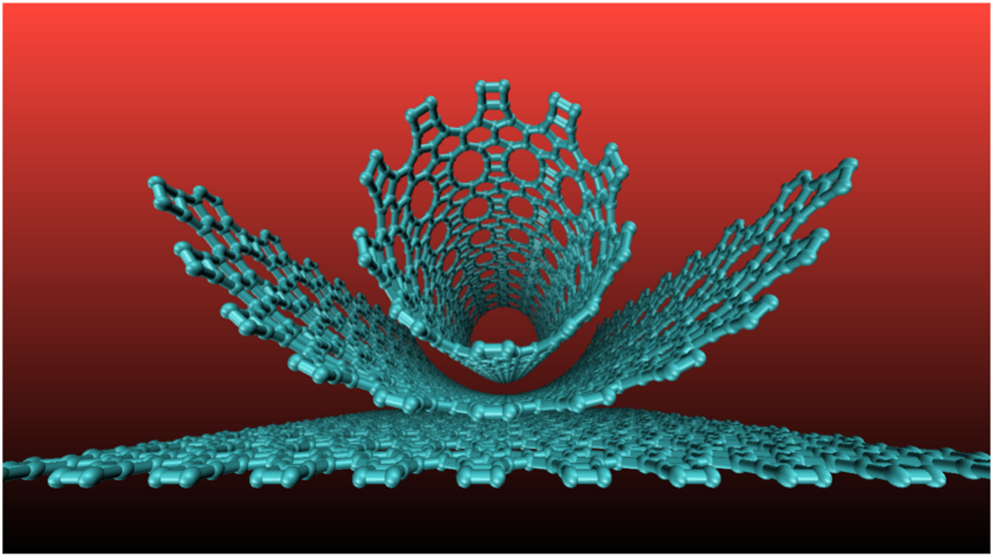

Tetra–Penta–Deca–Hexa graphene (TPDH) is a new two-dimensional (2D) carbon allotrope with attractive electronic and mechanical properties. It is composed of tetragonal, pentagonal, decagonal and hexagonal carbon rings. When TPDH graphene is sliced into quasi-one-dimensional (1D) structures such as nanoribbons, it exhibits a range of behaviors, from semimetallic to semiconducting. An alternative approach to achieving these desirable electronic properties (electronic confinement and nonzero electronic band gap) is the creation of nanotubes (TPDH-NTs). In the present work, we carried out a comprehensive study of TPDH-NTs combining Density Functional Theory (DFT) and classical reactive Molecular Dynamics (MD). Our results show structural stability and a chiral dependence on the mechanical properties. Similarly to standard carbon nanotubes, TPDH-NT can be metallic or semiconductor. MD results show Young’s modulus values exceeding 700 GPa, except for nanotubes with very small radii. However, certain chiral TPDH-NTs (*n*, *m*) display values both below and above 700 GPa, particularly for those with small radii. Analysis of the evolution of von Mises stress and the distribution of C–C bond angles and lengths throughout the stress–strain process indicates the important role of tetragonal, pentagonal, and hexagonal rings for the mechanical response of TPDH-NTs. Tetragonal and pentagonal rings provide a rigid mechanical framework for TPDH-NTs (*n*, 0), whereas pentagonal and hexagonal rings provide TPDH-NTs (0, *n*) with greater flexibility.

## Introduction

Graphene stands out as being presently one of the most investigated materials due to its unique electronic and mechanical properties and potential wide applications, including medicine, electronic devices, agriculture, etc.^1−3^ The distinctive feature of graphene lies in its two-dimensional topology, where sp^2^ covalently bond carbon atoms in hexagonal rings form a triangular lattice, resulting in high resistance to mechanical deformations and high electronic mobility.^4,5^

Topologically, graphene nanoribbons (GNRs) and carbon nanotubes (CNTs) are structures formed by cutting and rolling up graphene layers. GNRs can be considered as quasi-one-dimensional (1D) carbon strips with finite-sized sp^2^ hybridization and well-defined (armchair or zigzag) edges.^6^ Depending on the GNR size, the electronic confinement can result in a nonzero electronic band gap, while graphene^7^ has a null band gap. CNTs depending on their chirality can exhibit semiconductor or metallic behavior.^8,9^

**Figure 1:**
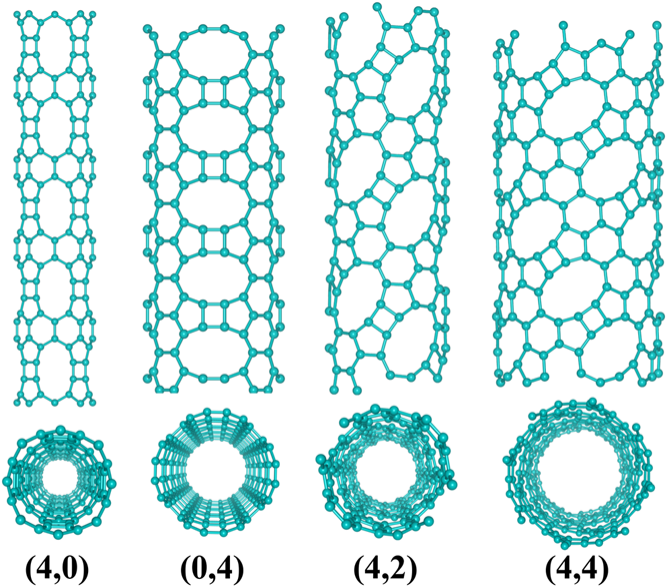
Representative examples (top and perspective views) of the types of TPDH nanotubes considered in this work. Zigzag ((4, 0) and (0, 4)), chiral ((4, 2)), and armchair ((4, 4)).

Graphene is just one member of the two-dimensional (2D) carbon allotrope family. There are hundreds of proposed structures, although only a few of them have been experimentally realized.^10^ 2D tetra–penta–hepta graphene (TPH-gr) is one of such allotropes that has recently been synthesized by the dehydrogenative C–C coupling of 2,6-polyazulene chains.^11^ TPH-gr is composed of C_4_, C_5_, and C_6_ carbon rings, having two phases exhibiting semiconductor properties with direct electronic band gaps of 2.704 and 2.361 eV, respectively.^12^ A closely related structure is the 2D tetra–penta–deca–hexa graphene (TPDH-gr). TPDH-gr is composed of C_4_, C_5_, C_10_, and C_6_ carbon rings (see Figure 1).

TPDH-gr was introduced by Bhattacharya and Jana.^13^ The proposed experimental approach involves using a fulvalene derivative as a precursor monomer. The dehydrogenation of these monomers can result in the formation of nanoribbons, which can then undergo simultaneous cycloaddition and further dehydrogenation to create the TPDH-gr structure.^13^

Density Functional Theory (DFT) calculations indicate that TPDH-gr exhibits a semimetallic behavior. However, when TPDH-gr is in the form of nanoribbons, confined in a quasi-1D structure, they present different electronic properties, such as a direct electronic band gap of ∼2.0 eV for one of the nanoribbon types (NR3).^13^

Recent studies on amorphous carbon materials, such as amorphous graphite and amorphous carbon nanotubes (a-CNTs), have shown how topological disorder affects their electronic and mechanical properties.^14,15^ Although TPDH-gr and TPDH nanotubes (TPDH-NTs) could appear to be carbon allotropes with topological defects, they exhibit regular and periodic atomic arrangements, clearly distinguishing them from amorphous allotropes.

It was reported that TPDH-gr has anisotropic mechanical properties, with values of stiffness constants of C_11_ = 244.48, C_22_ = 366.51, and C_12_ = 62.15 GPa·nm, indicating that TPDH-gr is stiffer when deformed along one direction than the other. It was also reported that TPDH-gr could be functionalized with hydrogen atoms, changing its electronic properties and creating anisotropy in its electronic transport.^16^ Additionally, through molecular dynamics simulations, Oliveira et al.^16^ showed that the carbon rings C_4_ are the sites of preferential hydrogenation (up to 80% at room temperature).

It was recently reported that TPDH-gr has a high theoretical specific capacity of 1116 mAh/g, suggesting its potential application as an anode in lithium-ion batteries (LIBs).^17^ Wu et al.^18^ studied the curvature effect of KT graphene on the performance of potassium-ion batteries (KIBs). They found that the internal tension generated during the formation of nanotubes from KT graphene positively affects the surface activity, enhancing both the diffusion and adsorption of potassium ions.^18^ In this sense, the TPDH-NTs would be attractive structures for such applications.

The aforementioned studies illustrate the dependence of electronic and mechanical properties on the types of carbon rings that comprise these allotropes. This is clearly evident in the cases of TPH-gr and TPDH-gr, which exhibit distinct electronic properties. Furthermore, electronic confinement within one-dimensional (1D) structures, such as nanoribbons and nanotubes, also introduces significant changes in the electronic and mechanical properties that can be exploited in different technological applications, such as in batteries.

Although nanoribbons of diverse carbon allotropes have been extensively investigated, the current literature still lacks such studies on the investigation of their corresponding nanotubes.^19−23^

In this work, we investigated the electronic and mechanical properties of TPDH-NTs through DFT and classical reactive MD simulations. The TPDH-NTs considered in this work include zigzag ((*n*, 0), (0, *n*)), armchair (*n*, *n*), and some chiral (*n*, *m*) nanotubes. The DFT simulations were used to obtain the electronic band structures and their corresponding density of states to determine the semiconductor or metallic nature of the nanotubes. MD was used to obtain the stress–strain curves and some elastic properties such as Young’s modulus (*Y*_M_), ultimate strength (US), and fracture limit (FL) values. Furthermore, the deformation mechanisms were investigated to address the relative importance of the different carbon rings in the TPDH-NT mechanical properties. Finally, the Young’s modulus of the TPDH-NTs was calculated using DFT to support the results obtained from molecular dynamics simulations.

## Computational Methods

### Modeling of TPDH-NTs

**Figure 2:**
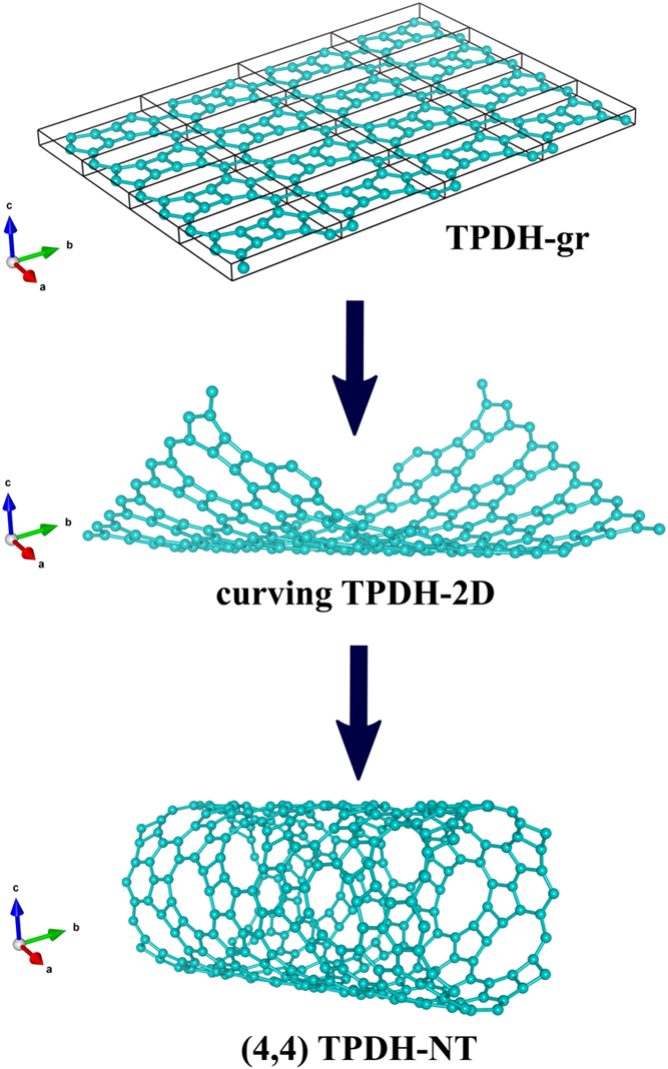
Representative scheme of generating the (4, 4) TPDH nanotube. Top: TPDH-gr unit cells were replicated for *n* = 4 and *m* = 4, where *n* and *m* are the chiral indices of the obtained TPDH nanotubes. Middle: TPDH-gr curving process. Bottom: Perspective view of formed TPDH-(4, 4) nanotube. The preservation of the TPDH-gr native carbon rings ((C_4_,C_5_,C_6_,C_10_)) can be observed.

As mentioned above, TPDH-gr is composed of tetragonal (C_4_), pentagonal (C_5_), hexagonal (C_6_), and decagonal (C_10_) rings, as shown in Figure 2. The TPDH-gr primitive vectors of the unit cell (*p⃗* = *ma⃗* + *nb⃗*) are also shown for the *m*, *n* = 4 case.

To create the TPDH-NTs models, we used the Cif2Tube code,^24^ which uses the crystallographic information (*.cif files) for generating the corresponding nanotube or nanoscroll-type systems. Cif2Tube applies the following transformation operations to the atomic positions of TPDH-gr in order to generate a nanotube-like system^24^
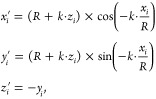
1

where *x*_*i*_, *y*_*i*_, and *z*_*i*_ are the TPDH-gr coordinates of the *i*th atom, *R* is the radius of the TPDH-NT, *k* is a dimensionless parameter that can take the values of 1 or −1, and *x*_*i*_^′^, *y*_*i*_^′^, and *z*_*i*_^′^ are the coordinates of the *i*th atom in the created nanotube system. Designation of the external surface is achieved using the value of *k* = [(*ma*, *nb*) × *L⃗*] · *c⃗*, where the vector *L⃗* is perpendicular to (*ma⃗*, *nb⃗*), and *c⃗* is a vector parallel to *z⃗*. If the value of *k* is positive, then the top surface becomes the external surface of a nanotube or a nanoscroll and vice versa.

We have considered four types of TPDH-NTs: zigzag, inverse zigzag, armchair, and chiral, which can be seen in Figure 1.

### *Ab Initio* Simulations

In this work, we carried out DFT^25,26^ simulations using the SIESTA code^27,28^ to investigate the structural stability and electronic properties of TPDH-NTs. Kohn–Sham orbitals were expanded using a double-ζ basis set composed of numerical pseudoatomic orbitals of finite range enhanced with polarization orbitals. A GGA-PBE type exchange and correlation functional was used, and to define the cutoff radii of the basis function, a common atomic confinement energy displacement of 0.02 Ry was applied, while the real-space grid fineness was determined by a mesh cutoff of 350 Ry.^29^ Regarding the exchange-correlation potential, the generalized gradient approximation was chosen,^30^ and pseudopotentials were modeled within the conservative Troullier–Martins^31^ norms in the Kleinman–Bylander factorized form.^32^

The structural models of TPDH-NTs generated using Cif2Tube software were fully relaxed, achieving residual forces below 0.01 eV/Å. This relaxation process resulted in a decrease in the total energy of the TPDH-NTs, primarily due to the redistribution of internal stresses and the adjustment of C–C bond lengths. We adopted a convergence criterion where self-consistency is achieved when the maximum difference between the output and the input of each element of the density matrix is less than 10^–4^ eV. Periodic boundary conditions were imposed, and a vacuum region of approximately 20 Å was added along the *x* and *y* directions, which are perpendicular to the axis of the nanotube (*z* direction). This ensures that the periodic images of the nanotube do not interact with each other and prevent spurious interactions.

We have employed a 1 × 1 × 8 *k*-point Monkhorst–Pack^33^ grid in our DFT calculations, as this configuration is well-suited for tubular systems. Given the one-dimensional periodicity of the TPDH-NTs along the *z*-axis, the 8 points along this direction ensure adequate sampling of the Brillouin zone to describe the electronic structure reliably. The 1 × 1 sampling along the transverse directions (*x* and *y*) is sufficient due to the effective isolation along these directions, reducing the computational cost while maintaining accuracy.

The cohesive energy *E*_coh_ (eV) was calculated for each TPDH-NT using the following equation
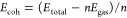
2where *E*_total_, *n*, and *E*_gas_ represent the total energy, number of carbon atoms, and energy of isolated carbon atoms, respectively. A more negative *E*_coh_ corresponds to a more stable structural configuration.

When a tubular structure is formed from a TPDH-gr, an internal strain is generated, which is specified as the curvature energy (*E*_curv_).^34^ This parameter is crucial for assessing the stability of the tube concerning the corresponding infinite flat sheet.^23,35^ The curvature energy *E*_curv_ is calculated as follows
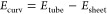
3where *E*_tube_ and *E*_sheet_ represent the total energy of the TPDH-NT and its corresponding TPDH-gr (sheet), respectively.

In the classical theory of elasticity, the curvature energy is expressed using the following formula^34,36^
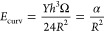
4where *Y* is the Young’s modulus, Ω is the area per carbon atom, *R* is the radius of the tube, and *h* is the thickness of the tube’s wall. Therefore, the approximate calculation of α can be quite useful to compare Young’s modulus of different TPDH-NTs.

The strain energy was obtained after TPDH-NTs stretching. The strain was calculated as ϵ_*z*_ = *L*/*L*_0_, where *L*_0_ and *L* are the relaxed and strained nanotube lengths, respectively. For each strain value, the TPDH-NTs nanostructure was fully relaxed until the maximum force component on each atom was less than 0.01 eV/Å. For each structural relaxation, the SCF convergence threshold for the electronic total energy was set at 10^–4^ eV.

To determine the elastic properties, the TPDH-NTs were treated as rolled membranes with thickness h equal to 3.35 Å and area equal to π*d*_t_*L*_0_ where *d*_t_ is the nanotube diameter. Therefore, the axial stress component σ_*z*_ is related to strain component ϵ_*z*_ as σ_*z*_ = (1/Ω)(∂*U*/∂ϵ_*z*_) where Ω = *L*_0_*π d*_t_*h* is the volume of the former nanotube membrane. Young’s modulus, *Y*, is estimated from the slope (dσ_*z*_/dϵ) of strain–stress curves in the linear regime.

### Molecular Dynamics Simulations

To investigate the mechanical properties of TPDH-NTs, we carried out classical reactive MD simulations at room temperature (*T* = 300 K) using a reactive force field (ReaxFF) implemented in the LAMMPS code.^37^ ReaxFF^38^ is an interatomic potential specifically designed to accurately model chemical bond breaking and formation. This reactive feature is essential for a correct description of mechanical properties beyond the linear regime, encompassing plastic deformation and fracture. Although other reactive potentials have been used in literature, such as the AIREBO-M.^39^ AIREBO-M is widely used to simulate the thermal and mechanical properties of carbon nanostructures,^40,41^ and it is known to require fine-tuning to adequately describe bond breaking. Figure S10 below shows a comparative analysis of the stress–strain behavior of the TPDH-NT (10,0) nanotube using ReaxFF and AIREBO-M. Our results demonstrate that the value of Young’s Modulus obtained using ReaxFF is closer to DFT one (see Figure S11 above). Based on that, we decided to use ReaxFF for our TPDH-NTs structures.

Before performing the simulation of the stress–strain process, each type of TPDH-NTs (zigzag (*n*, 0) and (0, *n*), armchair (*n*, *n*), and chiral (*n*, *m*)) was replicated (in the *z* direction) to a length of approximately 100 Å, in order to compare the different types of nanotubes and minimize the effects of finite size. Considering the number and size of the TPDH-NTs, carrying out this study with ab initio DFT molecular dynamics would be computationally very expensive.

To remove any initial stress before the stretching procedure, we subjected the nanotubes to a thermalization protocol within an isothermal–isobaric ensemble,^42^ with the pressure set to zero obtaining a minimum volume change of 1.0–3.0%. Throughout all MD simulations, the temperature was maintained at a constant 300K, controlled by a Nosé–Hoover thermostat,^43^ and the vacuum of 20 Å was maintained.

The stretching is induced by increasing the simulation box size along the periodic direction (the *z*-axis). The system dynamics were updated for every increase in 0.25 fs, for a total simulation time of 17.5 ps, maintaining a constant deformation/elongation rate of ∼3.5 × 10^–6^ fs^–1^. The elastic properties were evaluated using Young’s modulus values, estimated as (*Y* = dσ_*ii*_/dϵ_*ii*_), where (σ*_ii_*) represents the component of the virial tensor stress, and (ϵ_*ii*_) denotes the deformation along the axial direction *i*. The stress tensor is defined as
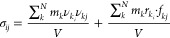
5

where *V* = *Ah* = *L*_0_π*d*_t_*h*, considering a hollow cylinder, is the volume of the TPDH-NT (zigzag, armchair, and chiral), *L*_0_ and *d*_t_ are the length and the diameter of the TPDH-NT, respectively, and *h* = 3.35 Å is the thickness value of the TPDH-NT structure. The stress distribution per atom in space was calculated using the von Mises stress tensor (σ_VM_), which is defined as^9,44,45^
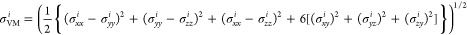
6

The average spatial distribution of the atomic stress evolution for all TPDH-NTs during the stretching process is also determined by using the von Mises stress tensor (eq 6). This stress tensor is specifically used to qualitatively evaluate the accumulation and/or dissipation of the stress values within the stretched structures.

## Results and Discussions

### Structural Stability and Electronic Properties

The arrangement of atoms in a TPDH-gr sheet is shown in Figure S1 in the Supporting Information. The lattice constants for the basic unit cell are defined as *a⃗*_1_ = 4.97 Å and *a⃗*_2_ = 7.02 Å. This basic unit cell contains 12 carbon atoms, categorized into four nonequivalent types denoted as 1, 2, 3, and 4, and the formation of C_6_ and C_10_ carbon rings can also be observed when the cell is replicated in the plane, as shown in Figure S1. Consequently, seven types of bonds are formed, including C_1_^*s*^ – C_1_^*s*^, C_1_^*L*^ – C_1_^*L*^, C_1_ – C_2_, C_2_ – C_3_, C_2_ – C_4_, C_3_ – C_3_, and C_4_ – C_4_ bonds. The C_1_^*s*^ – C_1_^*s*^ bonds denote connections between carbon atoms of type C_1_ with a bond distance of 1.45 Å, while the C_1_^*L*^ – C_1_^*L*^ bonds involve carbon atoms of type C_1_ with a larger bond length of 1.50 Å. The other types of bonds, C_1_ – C_2_, C_2_ – C_3_, C_2_ – C_4_, C_3_ – C_3_, and C_4_ – C_4_, exhibit lengths of 1.42, 1.45, 1.44, 1.37, and 1.48 Å, respectively. Our calculated lattice constants and bond length values are in good agreement with those reported in the literature.^13^

Our calculations showed a cohesion energy value of *E*_coh_ = −9.591 eV for TPDH-gr, just 0.348 eV larger than the cohesive energy for graphene. This result indicates that TPDH-gr has cohesive energy values in the same range as graphene and other 2D carbon allotropes, such as α -graphene, graphdiyne, and twin T-graphene.^10^

With the lattice parameters optimized for TPDH-gr, we created the four structural TPDH-NTs models (zigzag (*n*, 0) and (0, *n*), armchair (*n*, *n*), and chiral (*n*, *m*)) by rolling up the TPDH-gr sheets. As explained in the Computational Methods Section, the rolling was carried out using the Cif2Tube software,^24^ which uses the mathematical transformations shown in eq 1, the TPDH-NTs generated are described by the indices (*n*, *m*) that are analogous to the chiral indices of carbon nanotubes. Table 1 shows the number of atoms per cell, the diameter, and the length of these four TPDH-NTs models considered in this work.

**Table 1:** Structural Parameters of the Model TPDH-NTs. The Tube Chiralities Considered Here Are (*n*, 0), (0, *n*), (*n*, *n*), and (*n*, *m*)

chirality	*n*	atoms	diameter (Å)	length (Å)
(*n*, 0)	2	24	3.329	7.002
	3	36	4.849	7.018
	4	48	6.552	7.028
	5	60	7.966	6.991
(0, *n*)	2	24	4.226	4.935
	3	36	6.788	4.962
	4	48	8.736	4.953
	5	60	11.046	4.952
(*n*, *n*)	1	36	2.988	12.110
	2	72	5.760	12.175
	3	108	8.286	12.175
	4	144	10.809	12.181
(*n*, *m*)	(1, 2)	108	5.317	21.170
	(1, 3)	226	7.136	30.316
	(2, 1)	36	4.016	8.581
	(2, 3)	132	7.505	16.500
	(3, 1)	132	8.362	23.284
	(2, 4)	216	9.156	20.950
	(3, 2)	204	6.640	28.478
	(4, 1)	108	4.475	14.893
	(4, 2)	72	7.783	8.596
	(4, 3)	204	9.371	20.200

**Figure 3:**
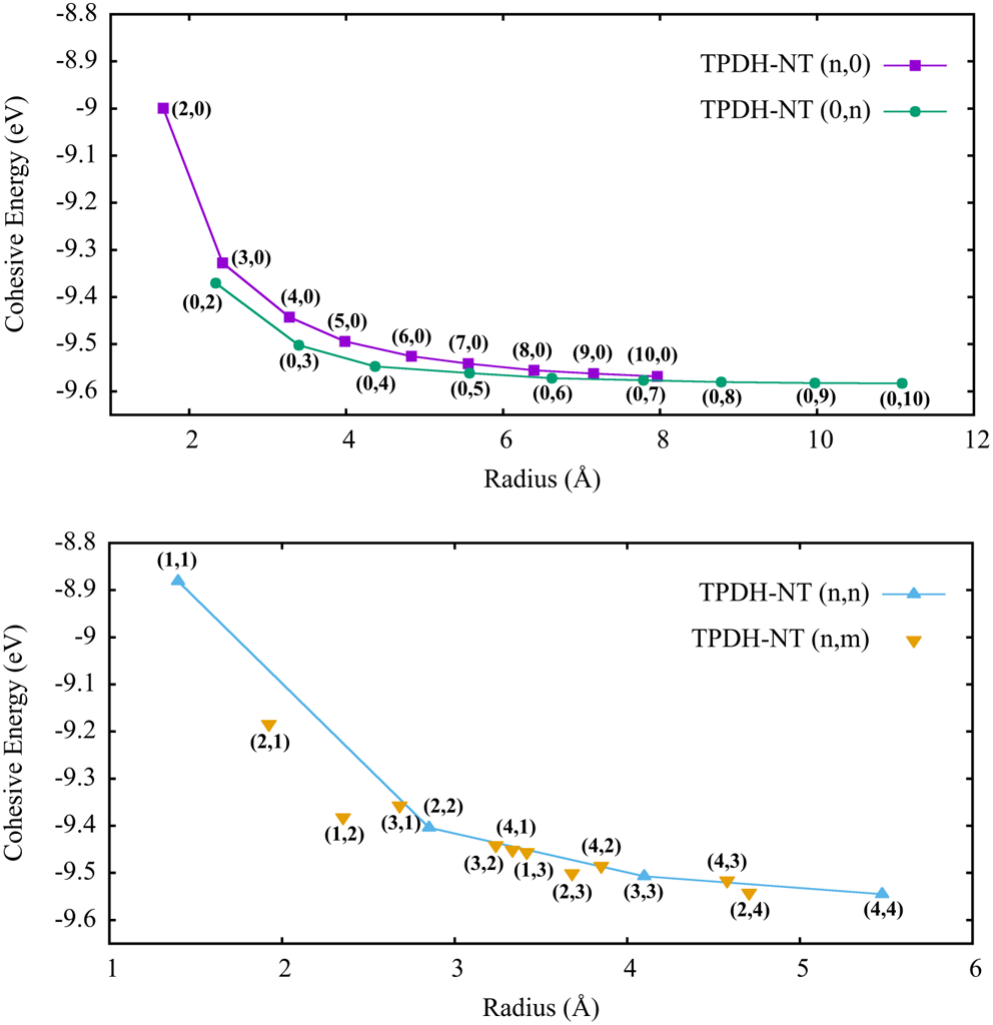
Cohesive energy (eV) values as a function of diameter (angstroms) for zigzag tubes (Top). Bottom: Armchair (*n*, *n*) and Chiral (*n*, *m*)TPDH-NTs.

In Figure 3, we present the values of the cohesive energy as a function of radius for the four TPDH-NT types considered in this work. Due to the high computational cost related to the number of atoms in the minimum unit cell, we have considered zigzag and inverse zigzag TPDH-NTs with a maximum value of the chiral index of *n* = 10, armchair TPDH-NTs with a maximum value of the chiral index *n* = 4, and some chiral TPDH-NTs.

As mentioned above, the cohesive energy is a measure of the structural stability of a system, more negative energies indicate more stable structures and whether the formation of that system is exothermic (thermodynamically favorable).^46,47^ As can be seen from Figure 3, for the zigzag, armchair, and chiral TPDH-NTs, the cohesive energy exhibits a strong dependence on the radius of the tubes, decreasing until it converges to the value of the cohesive energy of TPDH-gr.

The decrease in *E*_coh_ is related to the decrease in the surface strain of TPDH-NTs.^46,47^ For very large radius values, the energy differences between TPDH-NT and TPDH-gr tend to be zero, as expected. Based on the previous argument, it can be stated that the zigzag TPDH-NTs (0, *n*) have better structural stability because they have a larger intrinsic radius.

**Figure 4:**
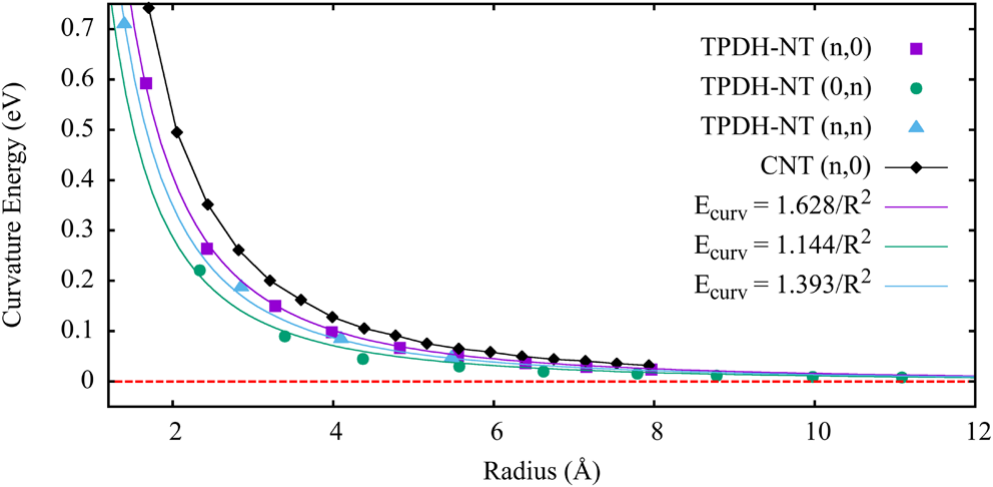
Curvature energy values (eV) for the different tube types. The solid lines represent the fitting of the relative energy using the function: *f*(D) = α /*r*^2^.

As discussed in the previous section, curvature energy *E*_curv_ is a very important parameter because it provides the energy difference between TPDH-NTs and TPDH-gr, and thus able to determine the effect of chirality and radius on the structural stability of TPDH-NT.^23,46^ In Figure 4, we present *E*_curv_ as a function of the radius for the different TPDH-NTs. We observe that all values are positive values, as expected, as the formation of TPDH-NTs is not spontaneous and requires energy to occur.^23,46^ It can also be observed that due to the larger intrinsic radius of the inverse zigzag TPDH-NTs (0, *n*), they require less energy to be rolled up, while the zigzag TPDH-NTs (*n*, 0) and armchair TPDH-NTs (*n*, *n*) show almost the same *E*_curv_ values.

In Figure 4, the fitted curves show a dependence on the radius of TPDH-NT of the following form: *f*(*r*) = α/*r*^2^. Therefore, we can use eq 4 to estimate and compare Young’s modulus for TPDH-gr.^34,35^

Based on eq 4, it can be deduced that α is the parameter that contains information on the elastic properties of the sheets that are used to create the nanotubes.^35^ Therefore, we performed a fitting of the *E*_curv_ as a function of the radius (*R*), to estimate the value of parameter α for the TPDH-NTs. The obtained values are α_(*n*,0)_ = 1.628, α_(0,*n*)_ = 1.144, and α_(*n*,*n*)_ = 1.393. These values indicate that the TPDH-gr has a higher Young’s modulus along the *x⃗* direction than *y⃗*. For TPDH-NTs, the longitudinal axis corresponds to *x⃗* for zigzag TPDH-NT, while *y⃗* corresponds to inverse zigzag TPDH-NTs. Therefore, we can deduce that zigzag TPDH-NTs exhibit a higher resistance to deformation than the other TPDH-NT types. Following this logic, we expect that the armchair TPDH-NTs are the next ordering type of NTs with the highest Young’s modulus, while the inverse zigzag type TPDH-NTs are the ones with the lowest resistance to deformation.

Additionally, in Figure 4, the *E*_curv_ of the zigzag carbon nanotubes (*n*, 0) is presented for different radius values to contrast the structural flexibility of CNTs and TPDH-NTs (*n*, 0) and TPDH-NTs (0, *n*). As can be seen from Figure 4, CNTs have a higher *E*_curv_ than TPDH-NTs (*n*, 0) and TPDH-NTs (0, *n*) for lower radius values (*r* < 6), while for radius values (*r* > 6), this difference in curvature energy is minimal. These results indicate that TPDH-NTs (*n*, 0) and TPDH-NTs (0, *n*) are more flexible than CNTs to form tubular *r* structures.

**Figure 5:**
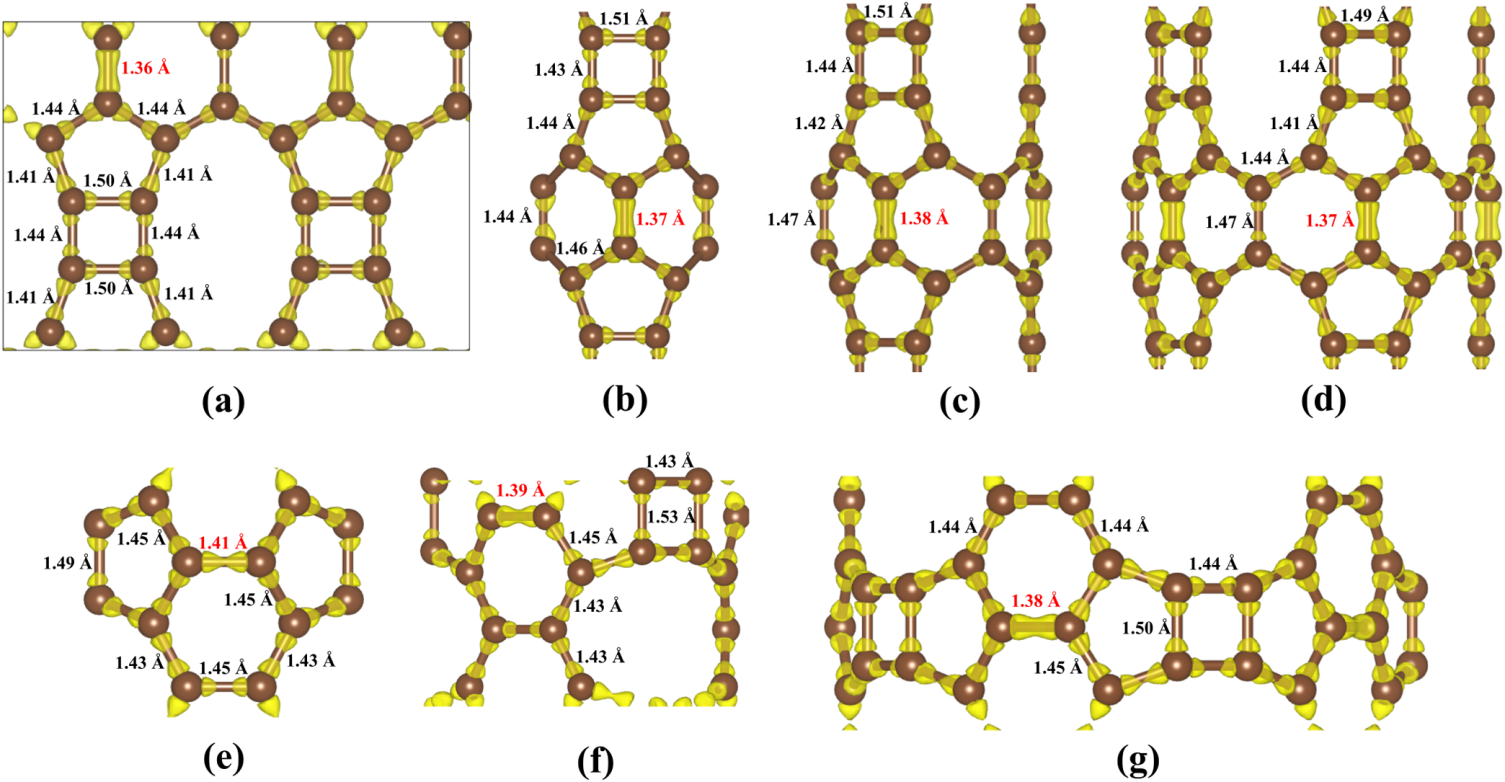
Total electron charge density (in yellow) of (a) TPDH-gr, (b) TPDH-NT (2,0), (c) TPDH-NT (3, 0), (d) TPDH-NT (5, 0), (e) TPDH-NT (0, 2), (f) TPDH-NT (0, 3), and (g) TPDH-NT (0, 5), at an isosurface value of 0.275 e/Å^3^. In all cases, a higher electron density is clearly observed along the hexagonal (C_6_) carbon rings.

In Figure 5, the total charge density and bond lengths for TPDH-gr and several TPDH-NTs (*n*, 0) and (0, *n*) are shown. As observed, TPDH-gr exhibits double bonds in the C_6_ rings, characterized by a higher localization of the charge density and a C–C bond length of 1.36 Å. These bond length values are characteristic of the sp^2^ hybridization, common in carbon structures like graphene. The TPDH-NTs also display carbon double bond like in the C_6_ rings, indicating *sp*^2^-like hybridization (formally, we can only refer to sp^2^ in planar structures), which implies partial delocalization of the π electrons, significantly contributing to the electronic stability of the structure.^35^

Figure S12 presents the orbital distributions for the first zigzag TPDH-NTs, highlighting the spatial arrangement of the π-type orbitals. The results indicate a greater delocalization in zigzag TPDH-NTs with (0, *n*) chirality compared to those with (*n*, 0) chirality, where the π-type orbitals are confined primarily to the C_5_ carbon rings. In contrast, in the (0, *n*) TPDH-NTs, the π-type orbitals are spread across the C_4_ and C_6_ carbon rings, enhancing electron delocalization and thereby increasing the electronic stability.^35^

Additionally, we infer that the longitudinal distribution of π-type orbitals along the axial direction in TPDH-NTs (*n*, 0) may contribute to greater stability against deformations as the electrons can be distributed more efficiently.

**Figure 6:**
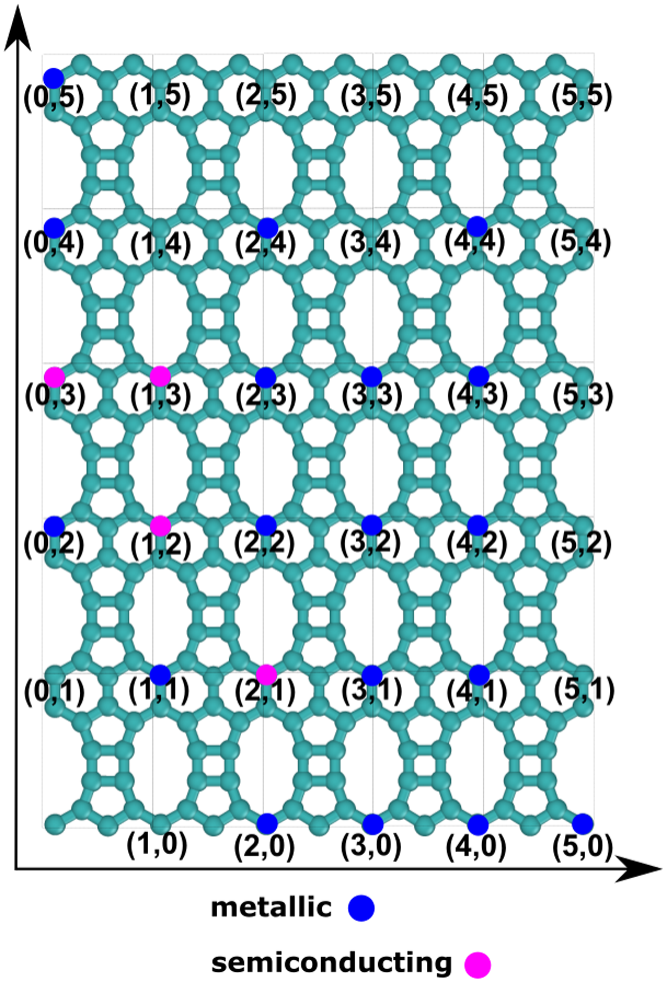
Summary of the electronic behavior (metallic or semiconducting) of the TPDH nanotubes considered in this work.

To investigate the electronic properties of the TPDH-NTs, the electronic band structure and density of states of the associated structures were calculated (see Figures S2–S4 of the Supporting Information). TPDH-gr has metallic behavior (no energy gap between the valence and conduction bands), while TPDH-gr nanoribbons exhibit an electronic band gap indicating semiconductor behavior.^13^ Due to the 1D confinement effect (nanoribbons) on the electronic properties of TPDH-gr, we can also expect to find a semiconducting behavior for the TPDH-NTs. Figure 6 summarizes the metallic or semiconductor behaviors of the different TPDH-NTs investigated here. As we can see, the TPDH-NTs with chiral numbers (1, 2), (2, 1), (0, 3), and (1, 3) are semiconductors, while the other TPDH-NTs have a metallic behavior. As we can see from Figure 3, these chiral semiconductor TPDH-NTs are those with small radius values (<2.75 Å). Another result of interest is the indirect electronic band gap of approximately 0.57 eV for the TPDH-NT (2, 1), which has a radius of ∼2.02 Å.

**Figure 7:**
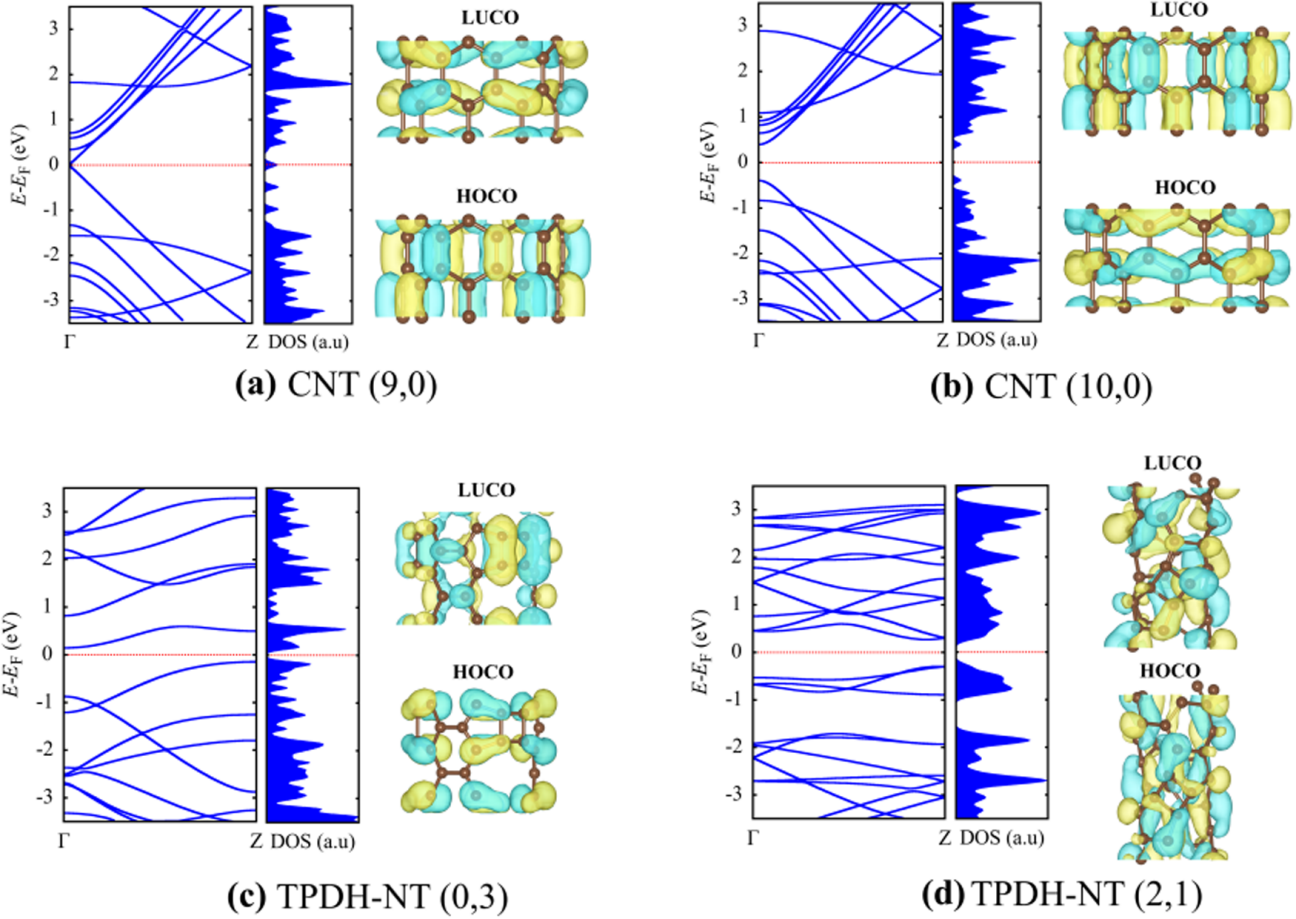
Electronic band structure and density of states of zigzag CNTs (9, 0) and (10, 0), and TPDH-NTs (0, 3) and (2, 1). The hoco and luco orbitals were plotted for each nanotube structure.

To better understand the effect of curvature on the electronic properties of TPDH-NTs, Figure 7 compares the electronic band structure, along with the HOCO and LUCO orbitals, of zigzag CNTs (9, 0) and (10, 0) with those of TPDH-NT nanotubes (0, 3) and (2, 1). In CNTs, the metallic or semiconducting behavior is defined by the rule of the difference in chiral indices (*n*-*m* = 3*i*), which states that if this difference is a multiple of 3, the nanotube will exhibit metallic behavior, while a nonmultiple leads to semiconducting behavior. According to this rule, CNT (9, 0) exhibits metallic properties, whereas CNT (10, 0) is a semiconductor. This distinction is clearly reflected in their band structures: the bands of CNT (9, 0) cross the Fermi level without breaks, while CNT (10, 0) displays an electronic band gap, confirming its semiconducting nature.

In contrast, the behavior of the TPDH-NTs is different. TPDH-NT (0, 3) is a semiconductor, as evidenced by its small electronic band gap. Meanwhile, TPDH-NT (2, 1) presents a significant band gap of 0.57 eV. These results align with the fact that TPDH-NTs, due to their unique structure and curvature, exhibit metallic or semiconducting behavior depending on chirality and size, although they do not follow the same explicit chirality-based rule as CNTs (this can be seen in Figure 6).

The HOCO and LUCO orbitals presented in Figure 7 for the CNTs and TPDH-NTs provide valuable information into their electronic properties. In CNT (9, 0), the HOCO orbital is delocalized along the longitudinal axis of the nanotube, facilitating electron mobility and thus explaining its metallic character. In contrast, the HOCO orbital in CNT (10, 0) is less delocalized and spatially more distributed along the tube radial parts. This leads to the opening of an electronic band gap and its semiconducting behavior. Similarly, in TPDH-NT (0, 3), the orbitals are predominantly along the radial parts, while the orbitals in other zigzag (0, *n*) TPDH-NTs (Figure S12) are π-type and aligned along the longitudinal axes, localized within the tetragonal *C*_4_ carbon rings. For zigzag (*n*, 0) TPDH-NTs, the π-type orbitals are localized in the *C*_5_ carbon rings.

Furthermore, the HOCO and LUCO orbitals in TPDH-NT (2, 1) show lower spatial delocalization due to the effects of curvature and chirality, contributing to its semiconducting behavior. The analysis of the electronic band structures and the distribution of HOCO and LUCO orbitals confirms that the semiconducting or metallic nature of TPDH-NTs, like CNTs, depends strongly on chirality and nanotube size. However, TPDH-NTs offer additional variety due to the distinctive structure of their carbon rings, which influences the distribution of π-type orbitals and the opening or closing of the electronic band gap.

### Mechanical Properties

As mentioned earlier, the study of the mechanical properties of TPDH-NTs was carried out through strain–stress analyses using classical reactive molecular dynamics (MD) simulations.^37^ In Figure S5, the stress–strain curves for TPDH-NTs with zigzag, armchair, and chiral tubes ((*n*, 0) and (0, *n*), (*n*, *n*), (*n*, *m*)) are presented, respectively. These results show that TPDH-NTs have distinct mechanical behaviors when under a stretching regime. In Tables S1–S4, some typical characteristics of the stress–strain curves are summarized, such as Young’s modulus Gpa (*Y*_M_), ultimate strength Gpa (US), and fracture strain % (FL).

Figure S5(a,b) show the stress–strain curves for the zigzag TPDH-NTs (*n*, 0) and (0, n); as can be seen, both types of NTs present different stress–strain profiles, with the values of ultimate strength (US) and fracture limit (FL) being the most distinct characteristic between both NTs, with the TPDH-NTs (0, *n*) having the highest US values.

**Figure 8:**
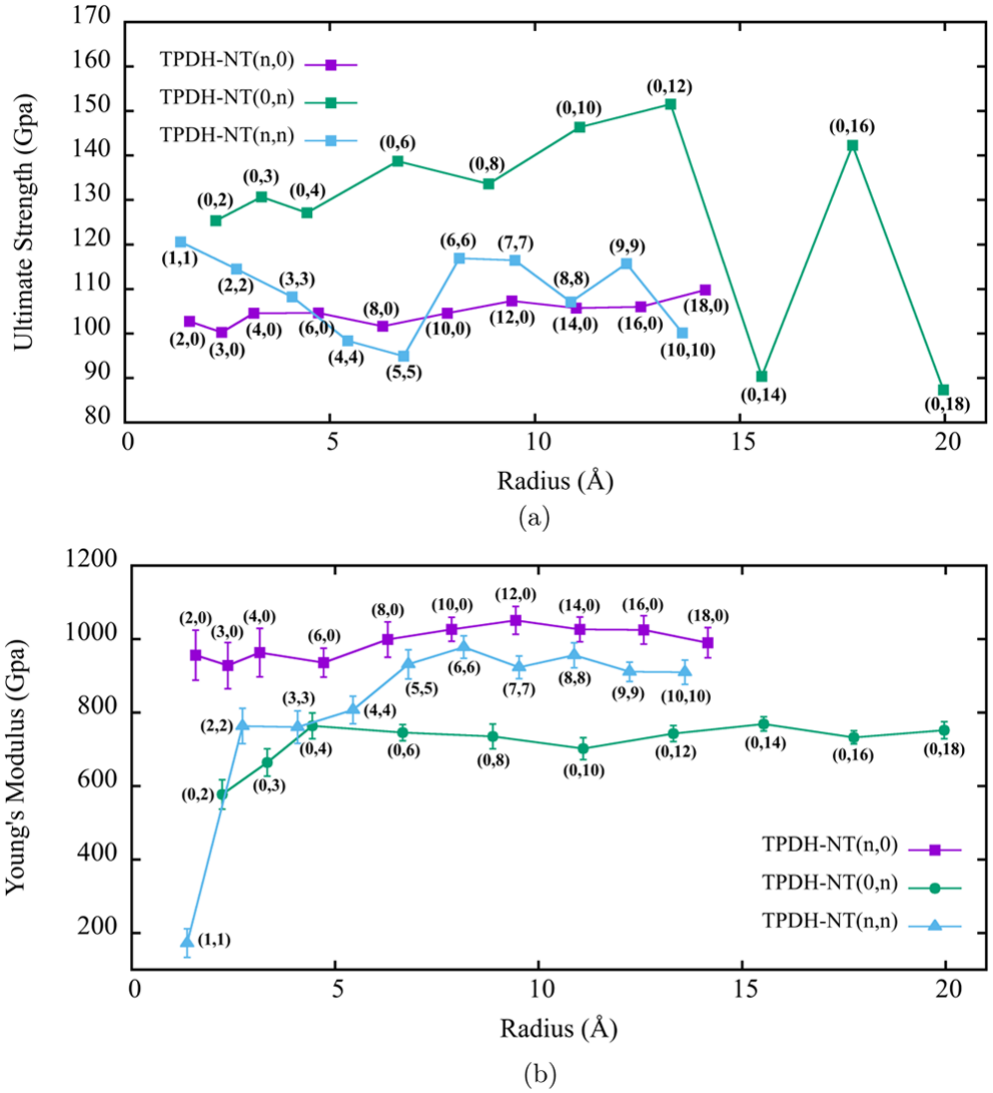
Ultimate strength (a) and Young’s modulus (b) values for TPDH-NTs zigzag (*n*, 0), inverse zigzag (0, *n*), and armchair (*n*, *n*).

From Table S1, we can see that there is a small variation in the ultimate strength (US) values among all zigzag TPDH-NTs (*n*, 0), as can also be seen in Figure 8a, the US values are plotted as a function of the diameter of the nanotubes. Furthermore, a clear correlation is observed between the FL and the nanotube radius. Consequently, nanotubes with larger radii are expected to exhibit FL values exceeding 20.8%. Conversely, the findings for TPDH-NTs with the chiral index (0, *n*) (refer to Table S2) indicate high US and FL values that scale with the nanotube radius, reaching a peak at 13.31 Å. Beyond this critical radius, larger than 13.31 Å, both US and FL tend to decrease and exhibit oscillations (Figure 8a).

Table S3 and Figure 8a present the FL and US values for the TPDH-NTs with armchair chirality (*n*, *n*). It is observed that the US and FL values decrease with the radius compared to the zigzag and inverse zigzag (*n*, 0) and (0, *n*) TPDH-NTs. However, for a radius value (6.80 < *r* < 12.23 Å), then for (*r* > 12.23 Å), the values of US and FL tend to increase.

On the other hand, Table S4 shows the values of US, FL, and YM for the chiral TPDH-NTs (*n*, *m*). Due to the chiral nature of these NTs, it is not possible to establish a direct relationship of these mechanical parameters with the radius of the NTs. However, the US values shown in Table S4 indicate that TPDH-NTs (*n*, *m*) have a wide range of US values with a maximum value for NT (3, 1) and a minimum value for NT (2, 3).

The Young’s modulus values for the TPDH-NTs (*n*, 0), (0, *n*), and (*n*, *n*) as a function of the radius of the NT are shown in Figure 8b. These results clearly show a dependence of the chirality on the elastic properties of the NTs studied. As described by the parameters α coming from the calculations of *E*_curv_, the TPDH-NTs with zigzag chirality (*n*, 0) are the stiffest NTs (with the highest value of *Y*_M_). On the other hand, despite being counterintuitive, TPDH-NTs with inverse zigzag chirality (0, *n*) are the NTs with the lowest value of *Y*_M_, indicating that a distribution of horizontal rings *C*_10_ affects negatively.

On the other hand, TPDH-NTs with armchair chirality (*n*, *n*) show modulus values of *Y*_M_ larger than TPDH-NTs (0, *n*) but lower than TPDH-NTs (*n*, 0), which is in accordance with the classical theory of elasticity. Therefore, we can conclude that the *Y*_M_ MD results are consistent with the DFT ones (which do not take into account the effect of the temperature).

**Figure 9:**
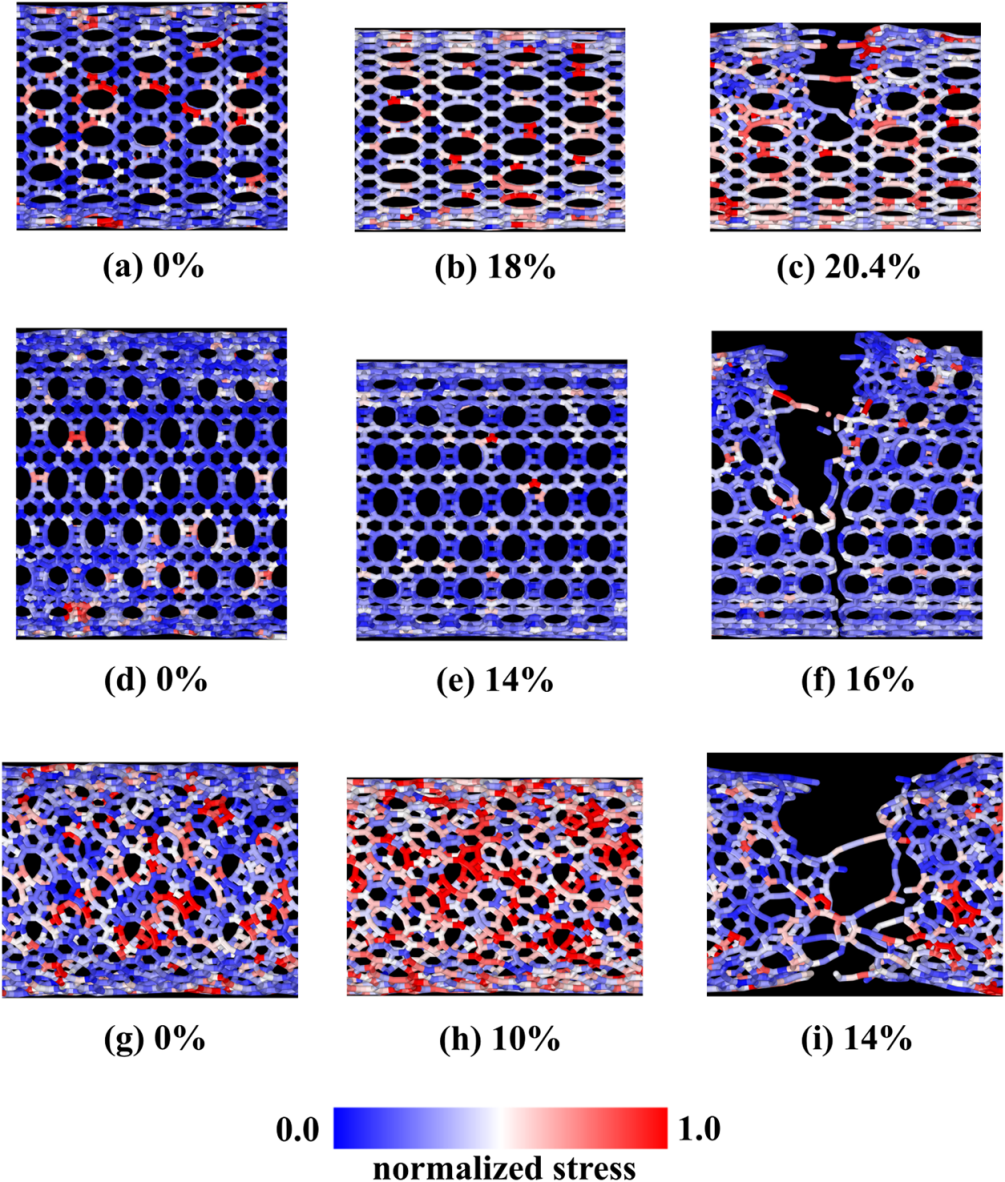
Representative MD snapshots showing the spatial distribution of von Mises stress for the TPDH-NT (zigzag, inverse zigzag, and armchair zigzag). (a–c) TPDH-NT (18, 0). (d–f) TPDH-NT (0, 18). (g–i) TPDH-NT (10, 10).

Besides obtaining the values of the mechanical parameters (*Y*_M_, US, and FL), understanding the TPDH-NT deformation mechanism is also important. Figure 9 shows the spatial distribution of the von Mises stress for the TPDH-NTs (18, 0), (0, 18), and (10, 10) for different strain values. These results help us visualize where the stress accumulates during the deformation process and thus estimate the effect of chirality (structure of the NTs) on these properties.

In the top part of Figure 9, the von Mises distribution of the TPDH-NT (18, 0) is shown for the strain of 0, 18, and 20.4%. For the value of 0%, residual stress is observed from the thermalization process, where apparently the stress is located in the tetragonal (C_4_) and pentagonal (C_5_) type carbon rings. On the other hand, for a value of ϵ = 18%, the stress is clearly located in the C_4_ and C_5_ rings. Finally, for ϵ = 20.4%, the fracture of the NTs is shown, where breaking of the bonds of the C_4_ rings and the formation of linear carbon chains (LACs) can be observed.

Figures S5 and S6 show the distribution and angle values of C–C bonds, respectively, of the TPDH-NT (18, 0) for a deformation of 0 and 15%. It is possible to observe how the initial angle distribution is affected by strain, shifting the initial angle peak values. The main changes in the angle values involve C_6_ and C_5_, indicating a decrease in the C bond angle values.

**Figure 10:**
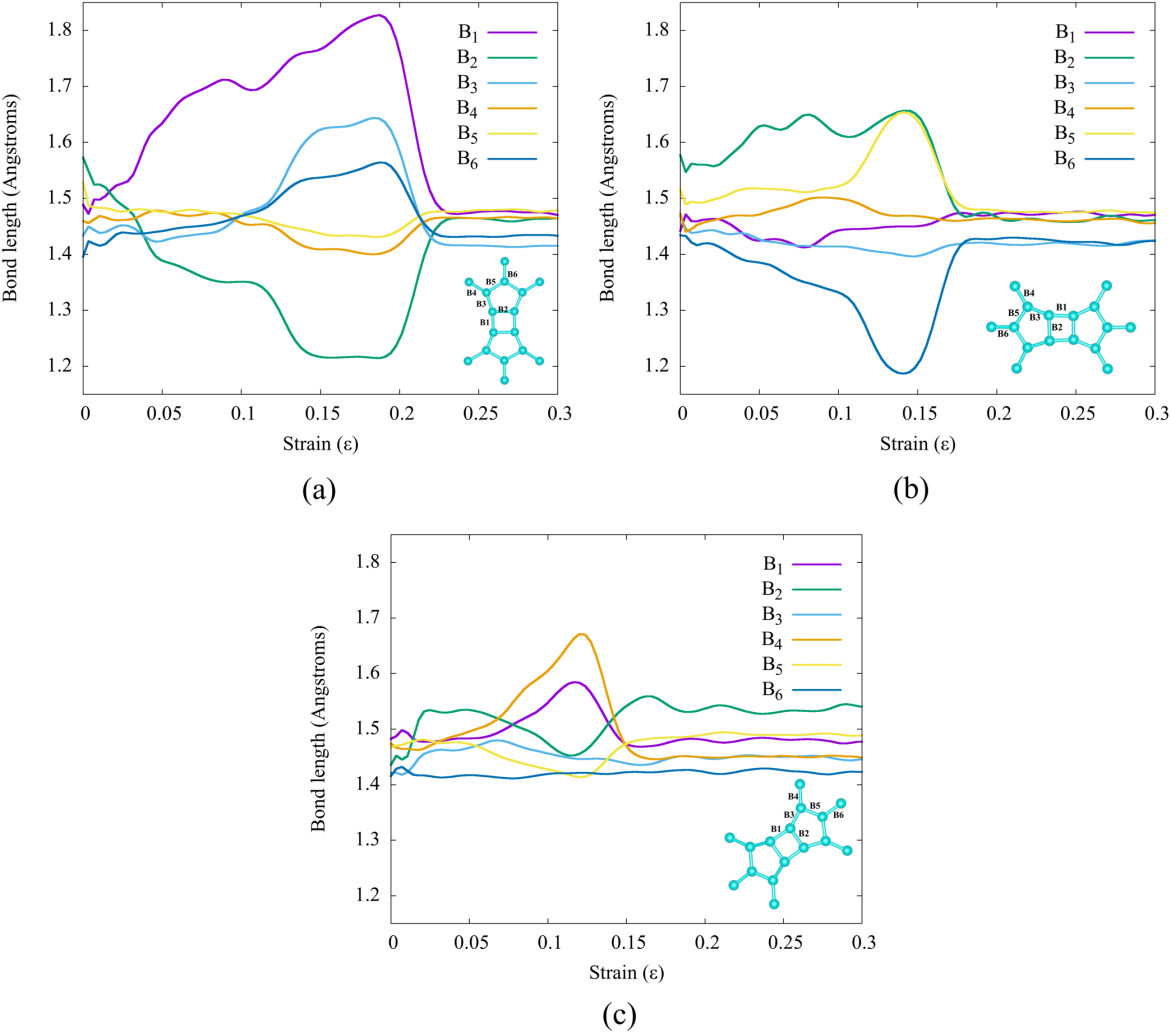
Bond length (C,C) values for (a) TPDH-NT (18, 0), (b) TPDH-NT (0, 18), and (c) TPDH-NT (10,10). The bonds B1, B2, B3, B4, B5, and B6 represent equivalent bonds for the TPDH-NTs.

In Figure 10a, C–C bond distances are presented as functions of strain values. The bonds B_1_ and B_2_ of the tetragonal ring C_4_ exhibit both stretching and compression, occurring in both the elastic and the plastic regions. In contrast, bonds B_3_ and B_6_ representing rings C_5_ and C_6_ only undergo stretching in the plastic region. Consequently, it can be inferred that the tetragonal C_4_ and pentagonal C_5_ rings are the regions where stress is accumulated. Thus, they play a crucial role in determining the mechanical properties of TPDH-NTs (*n*, 0).

Similarly, we can apply the aforementioned analyses to other zigzag TPDH-NTs with chirality (0, *n*) and armchair (*n*, *n*) configurations. Figure 9(d–f) present the distribution of von Mises stress for the NT (0, 18) at strain levels of ϵ = 0, 14, and 16%. In the case of NT (18, 0), residual stress from thermalization is observed. However, at a strain value of ϵ = 20%, distinctive patterns emerge in the C_5_ and C_6_ rings of NT (0, 18), suggesting that stress is accumulated in these regions, in contrast to NT (18, 0), where it is in the C_5_ and C_4_ rings. This finding is corroborated by the angle distribution analyses, as presented in Figures S5b and S6b, showing the angle deformation of the C_5_ and C_6_ rings. Additionally, the analyses of C–C bond lengths indicate stretching and compression in bonds B_5_ and B_6_, which are part of hexagonal carbon ring C_6_. Therefore, we can conclude that the carbon rings C_5_ and C_6_ present in the NT (0, 18) are responsible for this NT having small US and FL values because the carbon bonds in a pentagonal and hexagonal ring are weak due to their symmetry and hybridization.

Finally, the Von Mises stress for TPDH-NTs with armchair chirality (10, 10) is presented in Figure 9(g–i) for strain levels of 0, 10, and 14%. Notably, at a strain of ϵ = 10%, the stress is more uniformly distributed across the entire nanotube. The C–C angle distribution results (Figure S5(c)) reveal a more homogeneous profile without prominent peaks. Furthermore, the bond length analyses in Figure 10c demonstrate the stretching of C–C bonds occurring in almost all carbon rings, with only two unique peaks corresponding to the stretching of B_1_ and B_2_. Based on these findings, we can infer that TPDH-NTs exhibit larger elastic regions compared to other TPDH-NT nanotubes (*n*, 0) and (0, *n*), which display well-defined yield strength values in their stress–strain curves.

**Figure 11:**
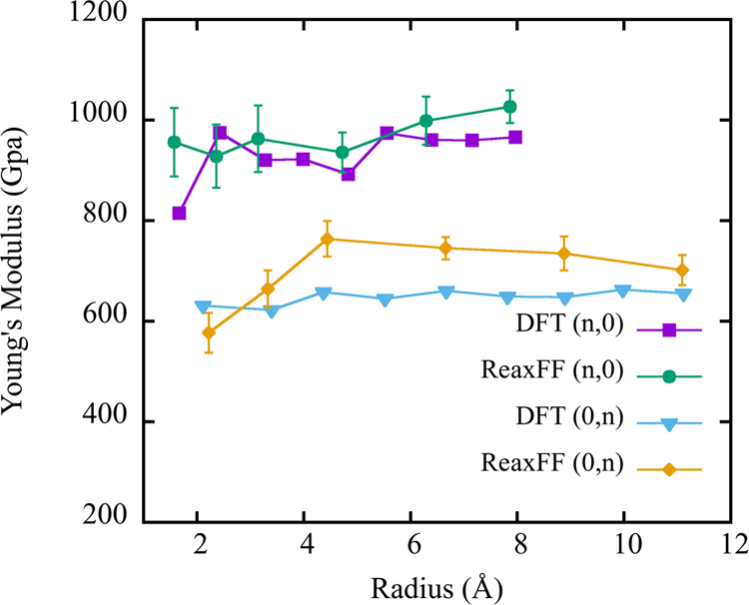
Comparison of Young’s modulus values obtained from DFT and molecular dynamics simulations for zigzag (*n*, 0) and (0, *n*) TPDH-NTs.

The stress–strain curves for (a) TPDH-NTs (*n*, 0) and (b) TPDH-NTs (0, *n*), calculated using density functional theory (DFT), are presented in Figure 11, where Young’s modulus values as a function of the radius for zigzag TPDH-NTs (*n*, 0) and (0, *n*) were calculated and presented together those obtained from molecular dynamics simulations. As we can see from the figure, the Young’s modulus values obtained by both methods are similar, particularly for larger radius values. This close correlation between the DFT and molecular dynamics results confirms the accuracy in describing the mechanical properties of the TPDH-NTs studied in this work. Additionally, in Figure S11, the stress–strain curves obtained by DFT are presented for the TPDH-NTs (*n*, 0) and (0, *n*), with the elastic deformation region (∼3%) presenting for most of the nanotubes.

**Figure 12:**
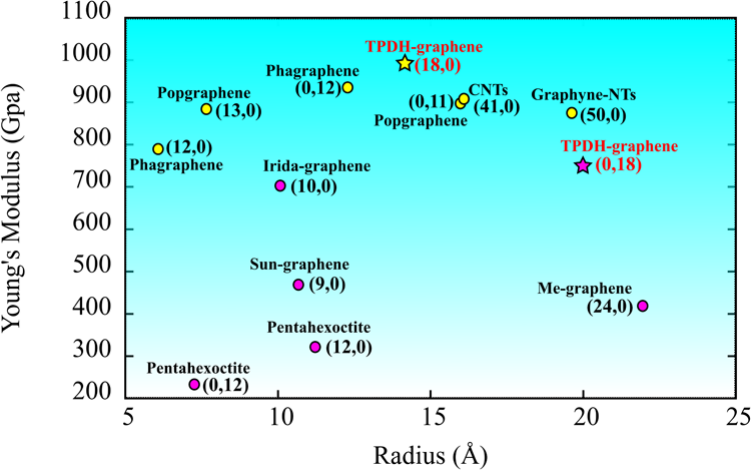
Comparison of Young’s modulus values for various zigzag ((*n*, 0) or (0, *n*)) nanotubes formed from different 2D carbon allotropes. All values were obtained from studies that employed computational dynamics as the primary method, and tubes with large radii were considered in order to compare Young’s modulus values in the asymptotic limit. The Young’s modulus values for TPDH-gr are indicated with a star symbol. The data were extracted from the following works: pentahexoctite,^48^ phagraphene,^22^ popgraphene,^21^ irida-graphene,^49^ sun-graphene,^49^ Me-graphene,^19^ and graphyne-NTs.^45^

In Figure 12, we compare Young’s modulus values for various zigzag nanotubes derived from different 2D carbon allotropes, including TPDH-NTs. These values were taken in the asymptotic limit. As we can see from the figure, TPDH-NTs rank among the most rigid nanotubes, with Young’s modulus values comparable to those of carbon nanotubes, popgraphene, phagraphene, and graphyne-NTs. This comparison contextualizes our findings and stresses the unique characteristics of TPDH-NTs in relation to other carbon nanotubes.

## Conclusions

In this study, we have investigated the electronic and mechanical properties of TPDH-NTs with chiralities (*n*, 0), (0, *n*), (*n*, *n*), and (*n*, *m*) using density functional theory (DFT) and reactive molecular dynamics simulations. The DFT results suggest structural stability, as evaluated by the calculation of the cohesive energy (*E*_coh_). The NT structural stability order is (0, *n*) > (*n*, 0) > (*n*, *n*) for radii values less than 5 Å. This ordering reflects the influence of the surface stress, which decreases as the tube diameter increases. Furthermore, the curvature energy calculations, *E*_curv_, revealed a chirality dependence in the elastic properties of the TPDH-NT, obtaining values for the α parameter, coming from classical elastic theory, in the following decreasing order: (*n*, 0) > (0, *n*) > (*n*, *n*). The chiral TPDH-NTs (*n*, *m*) were also investigated, presenting high structural and thermodynamic stability. Simultaneously, we analyzed the electronic band structures and density of states for all types of TPDH-NTs to determine the metallic or semiconductor nature of each nanotube, as summarized in Figure 6.

The results from molecular dynamics calculations are consistent with the DFT calculations and support the chirality dependence of the elastic properties of TPDH-NTs. Additionally, they indicate that the majority of TPDH-NTs with chiralities (*n*, 0), (0, *n*), and (*n*, *n*) are stiff with *Y*_M_ values exceeding 700 GPa, except for NTs with very small radii. However, certain chiral TPDH-NTs (*n*, *m*) display *Y*_M_ values both below and above 700 GPa, particularly for those with small radii. The analyses of the angle and C–C bond length distributions underscore the significance of the carbon rings (C_4_ and C_5_) and (C_5_ and C_6_) in influencing the mechanical response of zigzag TPDH-NTs (*n*, 0) and (0, *n*), respectively. In contrast, TPDH-NTs with chirality (*n*, *n*) exhibit a more uniform distribution in stress–strain profiles, effectively extending the elastic regime for these nanotubes.

Overall, the findings in this study provide valuable insights into the role of carbon rings in new 2D allotropes and their corresponding nanotubes, shedding light on the electronic and mechanical properties of these nanostructures. This deeper understanding could pave the way for future research and technological applications of these unique materials.

## Supporting Information Available

Structural information on TPDH graphene; electronic band and density of states calculations for zigzag ((*n*, 0), (0, *n*)), armchair (*n*, *n*), and various chiral (*n*, *m*) TPDH-NTs; graph of the stress–strain process; distribution of C–C bond angles; snapshots and structural values of TPDH-NTs during the stress–strain process; Young’s modulus for selected chiral TPDH-NTs; table of some mechanical properties data for TPDH-NTs; evolution of the transverse angle and C–C bond distance of the zigzag TPDH-NTs as a function of the chiral index (*n*); comparative stress–strain simulations for TPDH-NT (10, 0) for Reax and Airebo-M force field; stress–strain curves obtained from density functional theory calculations for zigzag TPDH-NTs; and molecular orbitals plots for zigzag TPDH-NTs (PDF)
