# Supplementary material for: Exploring the Electronic and Mechanical Properties of TPDH Nanotube: Insights from Ab Initio and Classical Molecular Dynamics Simulations
Source: ACS Omega. 2024 Dec 11;9(51):50225–36. doi: 10.1021/acsomega.4c05614 (PMC11683488; doi:10.1021/acsomega.4c05614)
Supplement: Supplementary file 1 — ao4c05614_si_001.pdf [file ao4c05614_si_001.pdf]

Exploring the Electronic and Mechanical Properties of TPDH-Nanotube: Insights from Ab initio and Classical Molecular Dynamics Simulations

Juan Gomez Quispe,[†] Douglas Soares Galvao,[‡] and Pedro Alves da Silva Autreto[*,†]

†*Electronic Structure and Atomistic Dynamics Interdisciplinary Group (GEEDAI), Center for Natural and Human Sciences(CCNH), Federal University of ABC (UFABC), Avenida dos Estados, 5001, 09210-580 Santo Andre 09210-170 , Sao Paulo, Brazil.*

‡*Applied Physics Department and Center for Computing in Engineering and Sciences, State University of Campinas, Rua Sergio Buarque de Holanda, 777, 13083-859, Campinas, Sao Paulo, Brazil.*

E-mail: pedro.autreto@ufabc.edu.br

Supporting Information Available

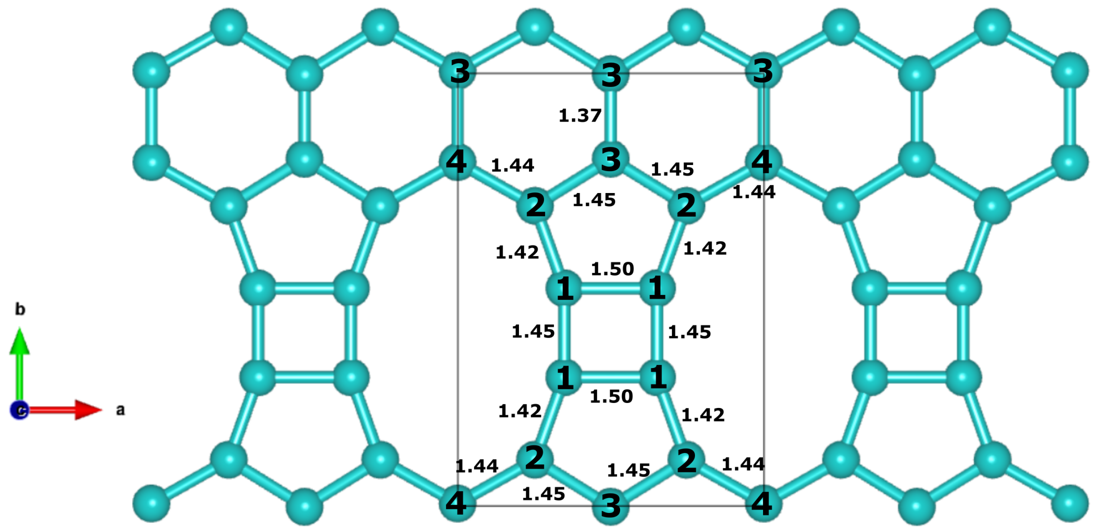

Fig. S1. Structural information of the TPDH-graphene, the bond distances are in Å.

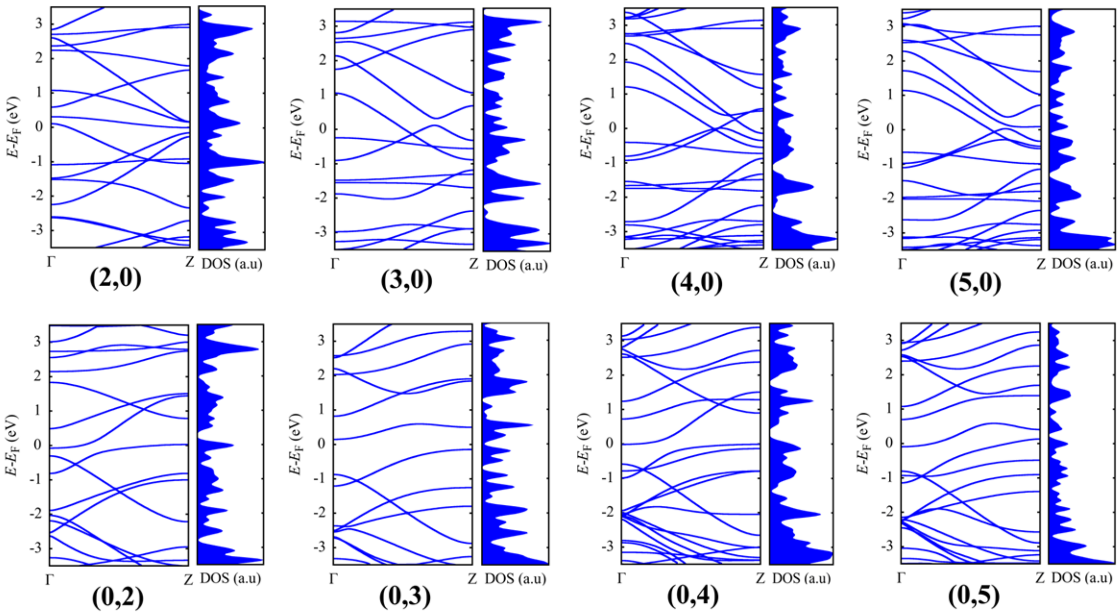

Fig. S2. Electronic band structure and density of states of TPDH nanotubes. Top: zigzag TPDH-NT (n,0). Bottom: inverse zigzag TPDH-NT (0, n).

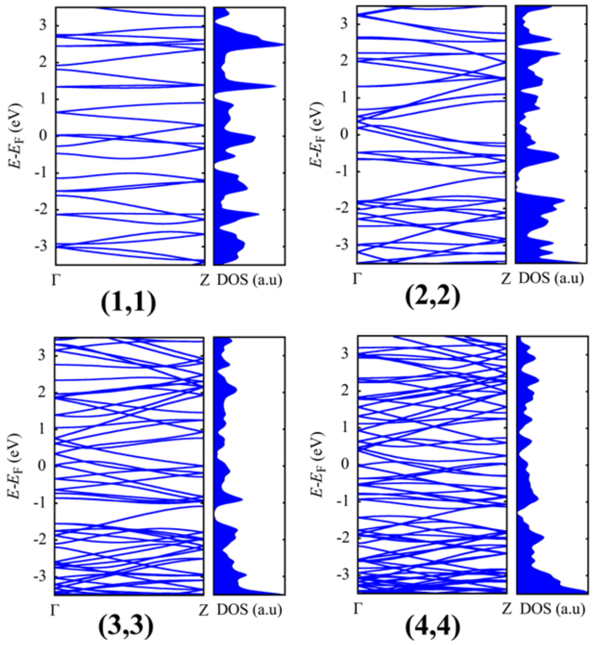

Fig. S3. Electronic band structure and density of states for armchair TPDH-NT (n,n).

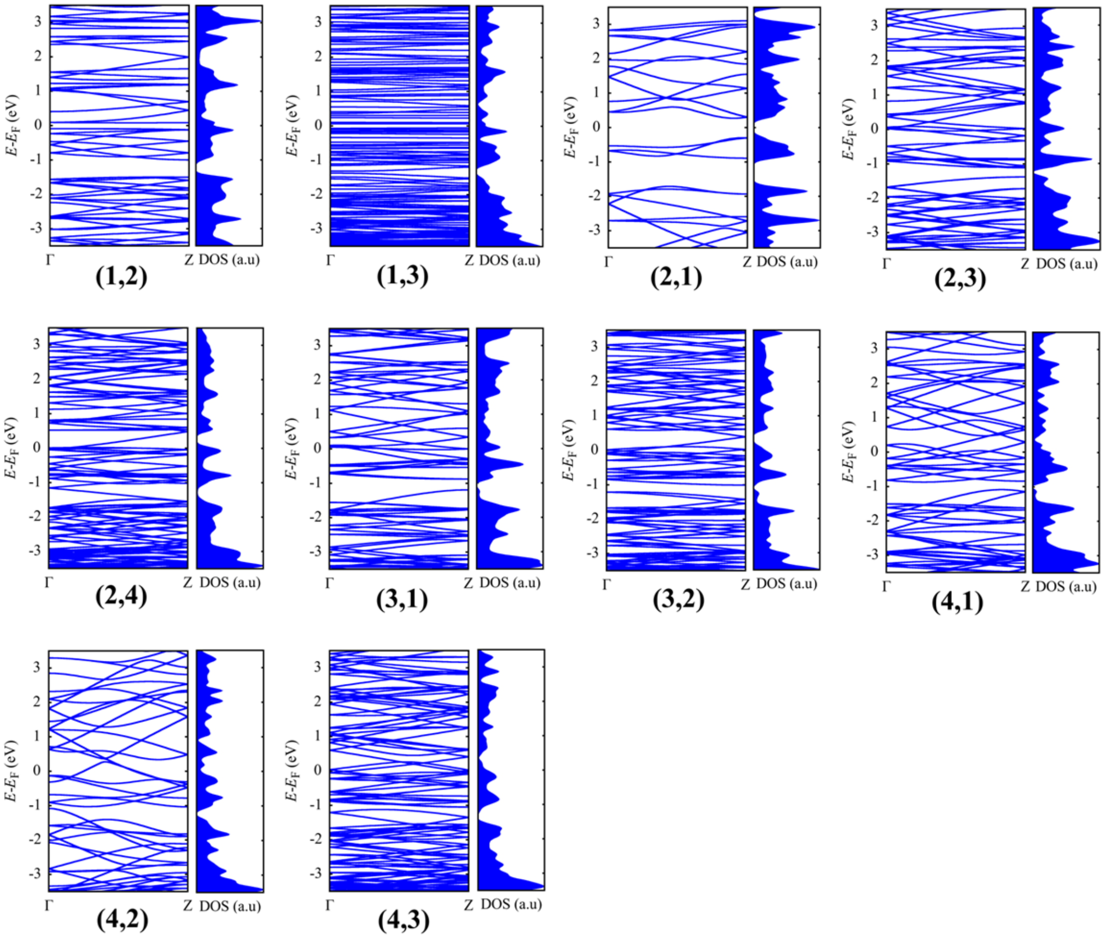

Fig. S4. Electronic band structure and density of states of chiral TPDH-NT (n,m).

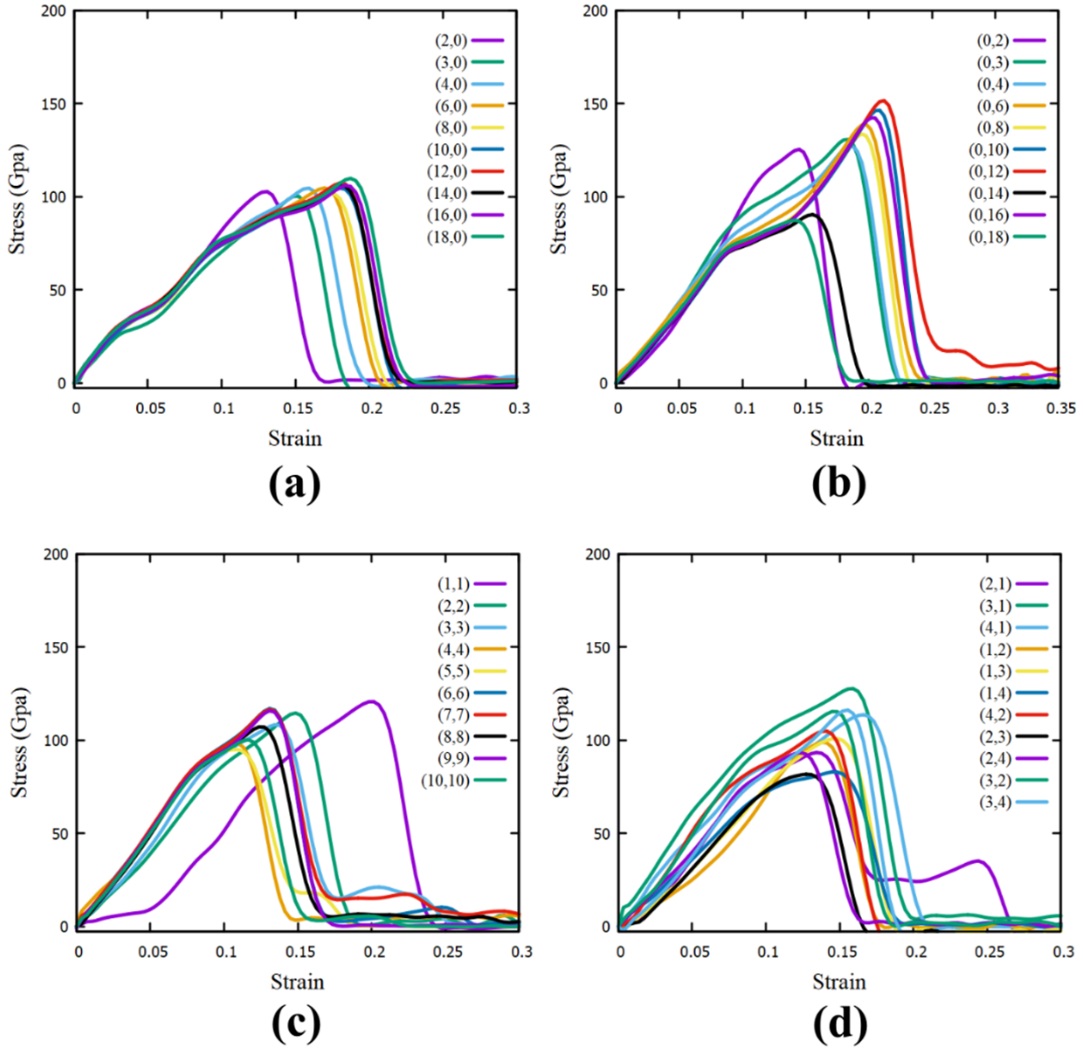

Fig. S5. Graph of the stress-strain process of TPDH nanotubes (zigzag, inverse zigzag, armchair, chiral). a) Zigzag TPDH-NT. b) Inverse zigzag-NT. c) Armchair TPDH-NT. d) Chiral TPDH-NT.

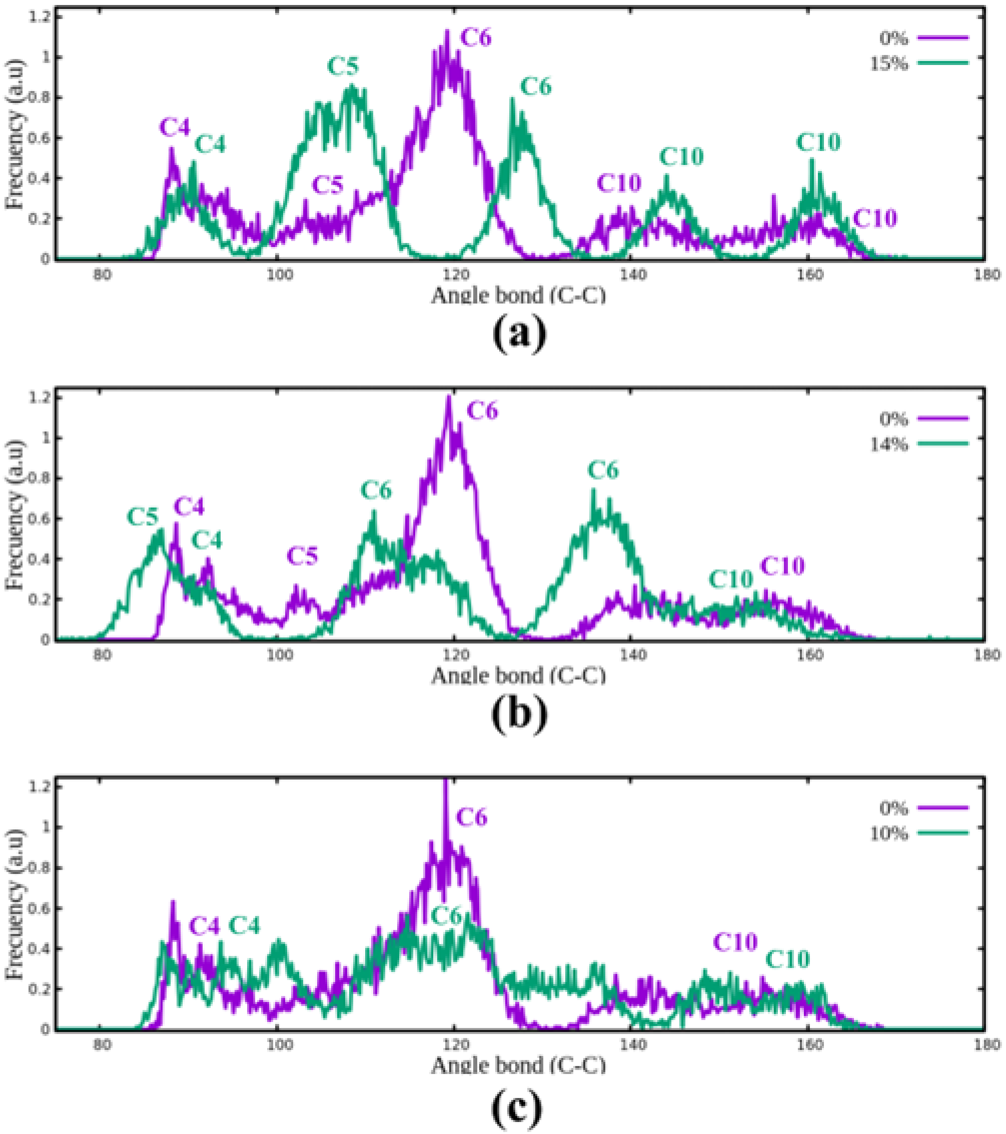

Fig. S6. Distribution of C-C bond angles for TPDH nanotubes (a) zigzag TPDH-NT (18,0), (b) inverse zigzag TPDH-NT (0,18) y (c) armchair TPDH-NT (10,10), for the initial state of zero strain, and 15%, 14%, 10%, respectively.

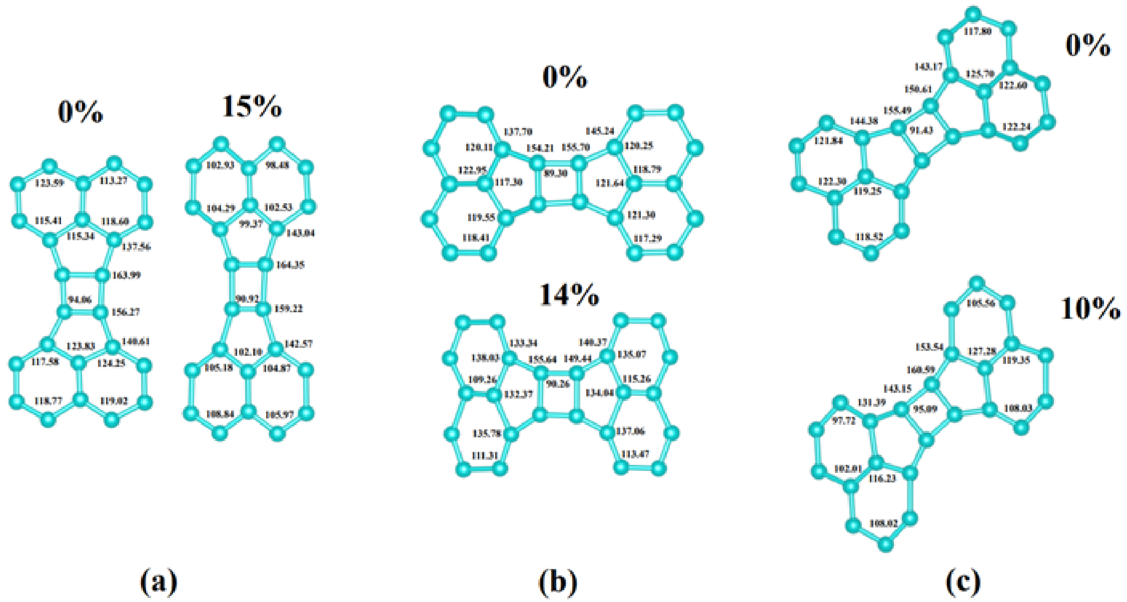

Fig. S7. Values of the C-C bond angles for the TPDH nanotubes for (a) zigzag TPDH-NT (18,0), (b) inverse zigzag TPDH-NT (0,18), and (c) armchair TPDH-NT (10,10), for a zero-strain state (0%) and non-zero strain (15 %, 14 %, 10 %).

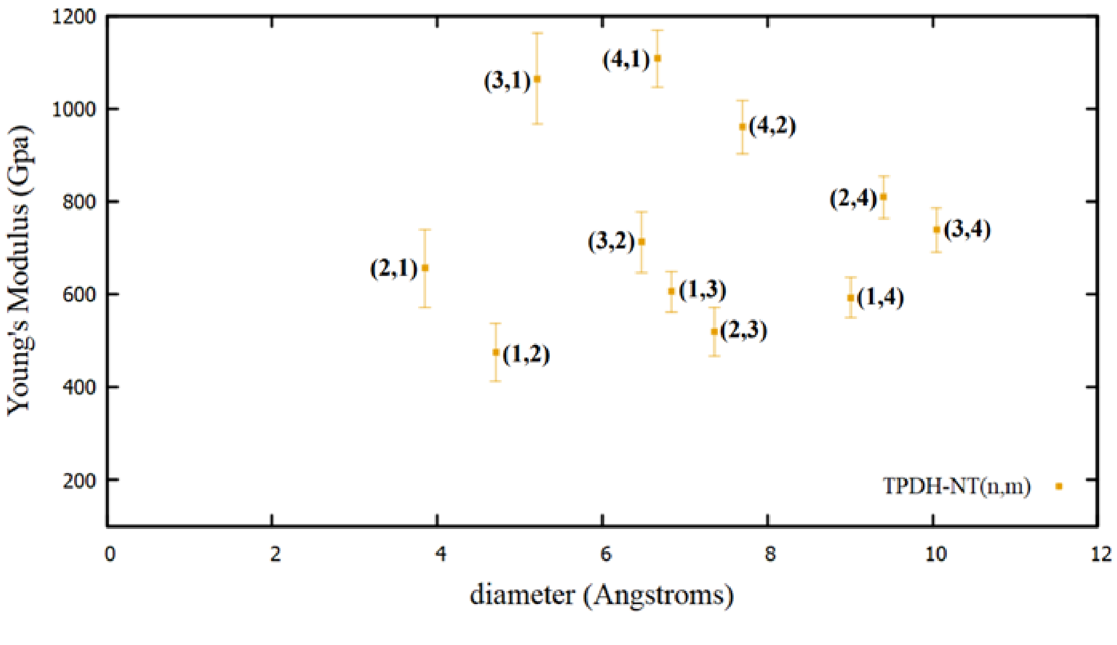

Fig. S8. Young's modulus values for some chiral TPDH nanotubes (n,m).

Table S1. Mechanical properties of Zigzag TPDH-NTs with different radius.

Zigzag-NTs	Radius(Å)	Young Modulus (Gpa)	Ultimate Strength (Gpa)	Fracture Strain (%)
(2,0)	1.57	956.34	102.752	14.6
(3,0)	2.36	928.20	100.261	16.7
(4,0)	3.14	963.22	104.577	17.6
(6,0)	4.72	935.98	104.637	18.7
(8,0)	6.29	999.01	101.659	19.2
(10,0)	7.86	1026.89	104.590	19.8
(12,0)	9.43	1051.41	107.352	20.2
(14,0)	11.01	1026.58	105.752	20.2
(16,0)	12.58	1025.16	105.997	20.8
(18,0)	14.15	990.40	109.780	20.8

Table S2. Mechanical properties of Inverse Zigzag TPDH-NTs with different radius.

Inverse Zigzag-NTs	Radius(Å)	Young Modulus (Gpa)	Ultimate Strength (Gpa)	Fracture Strain (%)
(0,2)	2.22	577.25	125.393	16.3
(0,3)	3.33	664.45	130.717	20.2
(0,4)	4.44	763.96	127.160	20.8
(0,6)	6.65	745.48	138.779	21.6
(0,8)	8.87	735.20	133.618	21.2
(0,10)	11.09	701.95	146.380	22.9
(0,12)	13.31	743.31	151.540	23.5
(0,14)	15.53	769.18	90.386	18.5
(0,16)	17.74	732.83	142.324	22.5
(0,18)	19.96	752.01	87.3326	16.8

Table S3. Mechanical properties of Armchair TPDH-NTs with different radius.

Armchair-NTs	Radius(Å)	Young Modulus (Gpa)	Ultimate Strength (Gpa)	Fracture Strain (%)
(1,1)	1.36	172.43	120.61	21.8
(2,2)	2.72	763.51	114.55	16.7
(3,3)	4.08	760.66	108.26	15.5
(4,4)	5.44	807.47	98.38	13.2
(5,5)	6.80	931.70	94.94	13.4
(6,6)	8.16	978.34	116.94	15.6
(7,7)	9.51	923.44	116.47	15.7
(8,8)	10.87	956.07	107.14	15.4
(9,9)	12.23	911.31	115.70	15.7
(10,10)	13.59	910.33	100.15	13.9

Table S4. Mechanical properties of Chiral (n,m) TPDH-NTs with different radius.

Chiral-NTs	Radius(Å)	Young Modulus (Gpa)	Ultimate Strength (Gpa)	Fracture Strain (%)
(2,1)	1.92	655.76	93.36	15.7
(3,1)	2.60	1064.19	127.70	17.7
(4,1)	3.33	1108.00	113.61	19.8
(1,2)	2.35	474.00	98.89	15.7
(1,3)	3.42	605.08	100.78	16.8
(1,4)	4.50	592.63	82.97	17.2
(4,2)	3.85	960.37	104.82	16.4
(2,3)	3.68	519.76	81.66	15.7
(2,4)	4.70	809.18	93.27	15.5
(3,2)	3.24	712.09	115.32	19.9
(3,4)	5.02	738.69	116.15	17.2

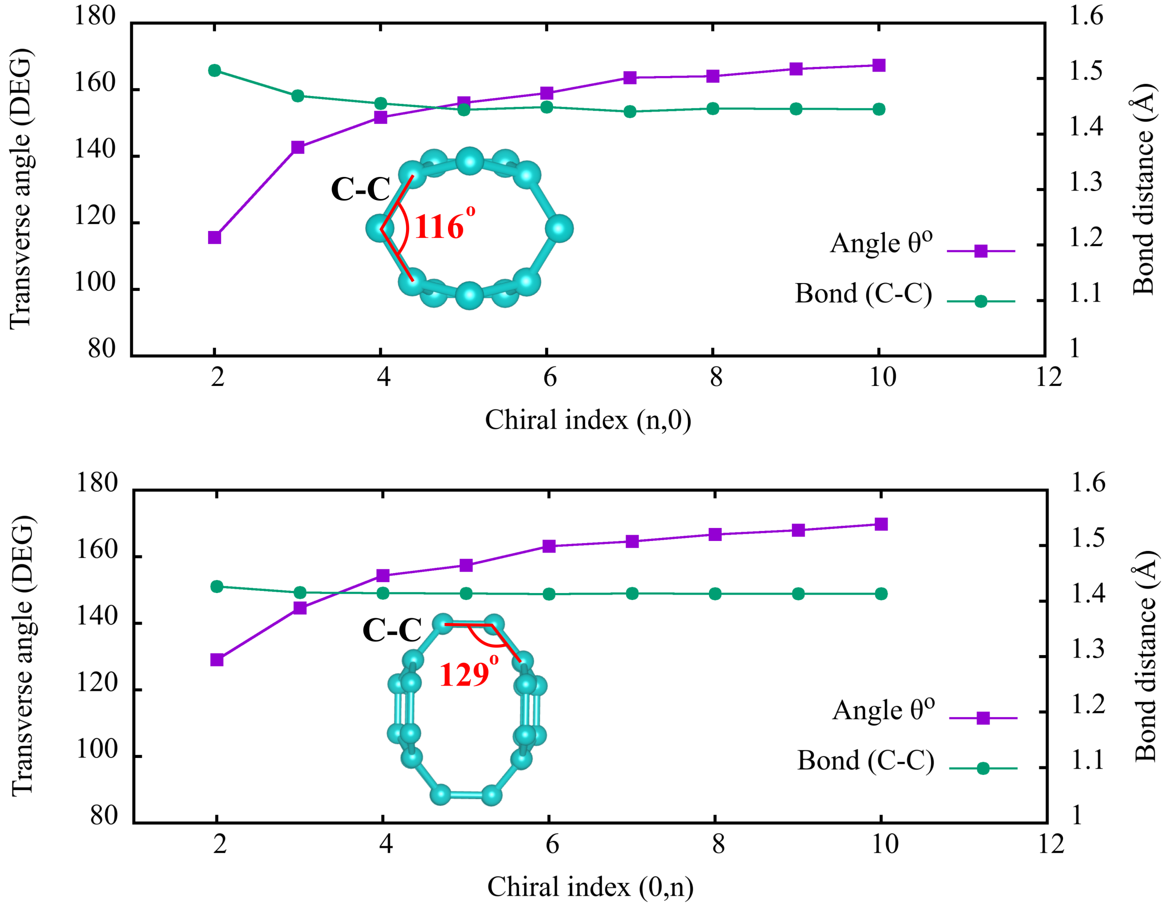

Fig. S9. Evolution of the transverse angle (and C-C bond distance) of the TPDH-NTs (n,0) and (0,n) as a function of the chiral index (n).

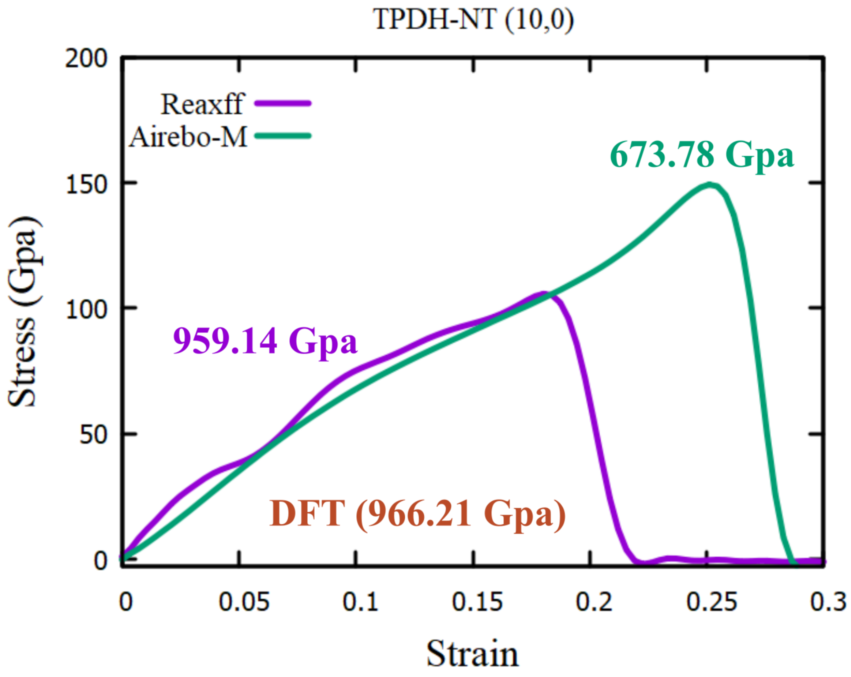

(a) Comparative simulations of force fields

Fig. S10. (a) Comparative stress-strain simulations for TPDH-NT (10,0) for Reax and Airebo-M force field.

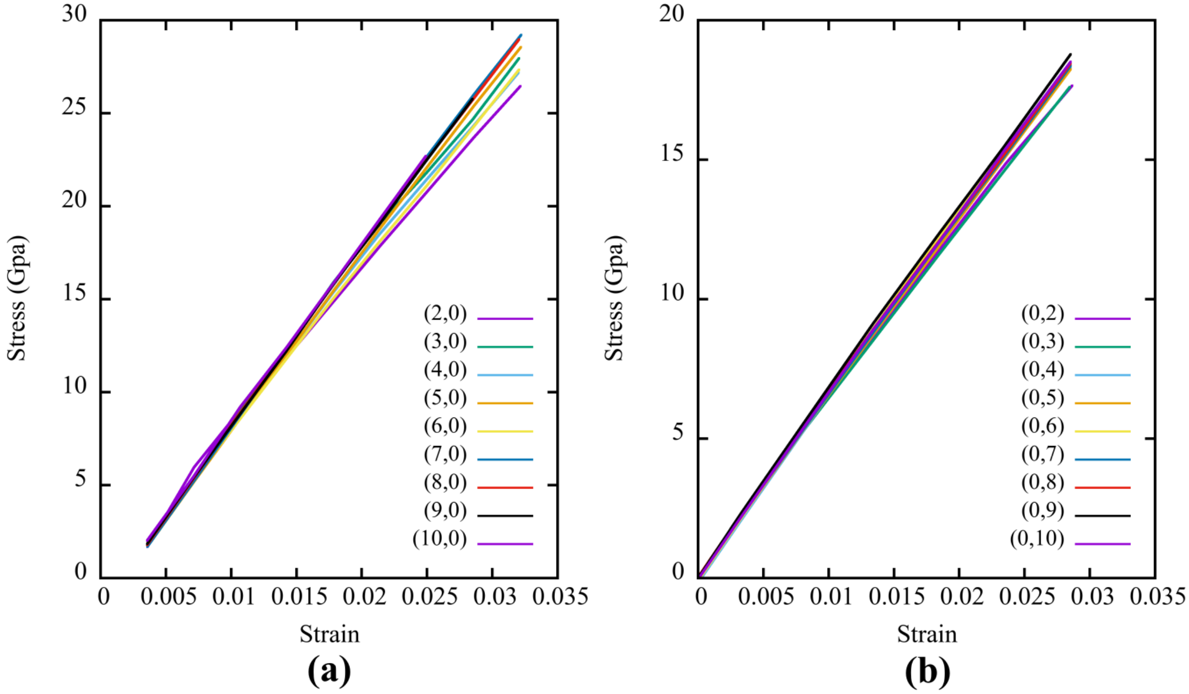

Fig. S11. Stress-strain curves for zigzag (a) TPDH-NTs (n,0) and (b) TPDH-NTs (0,n), calculated using Density Functional Theory.

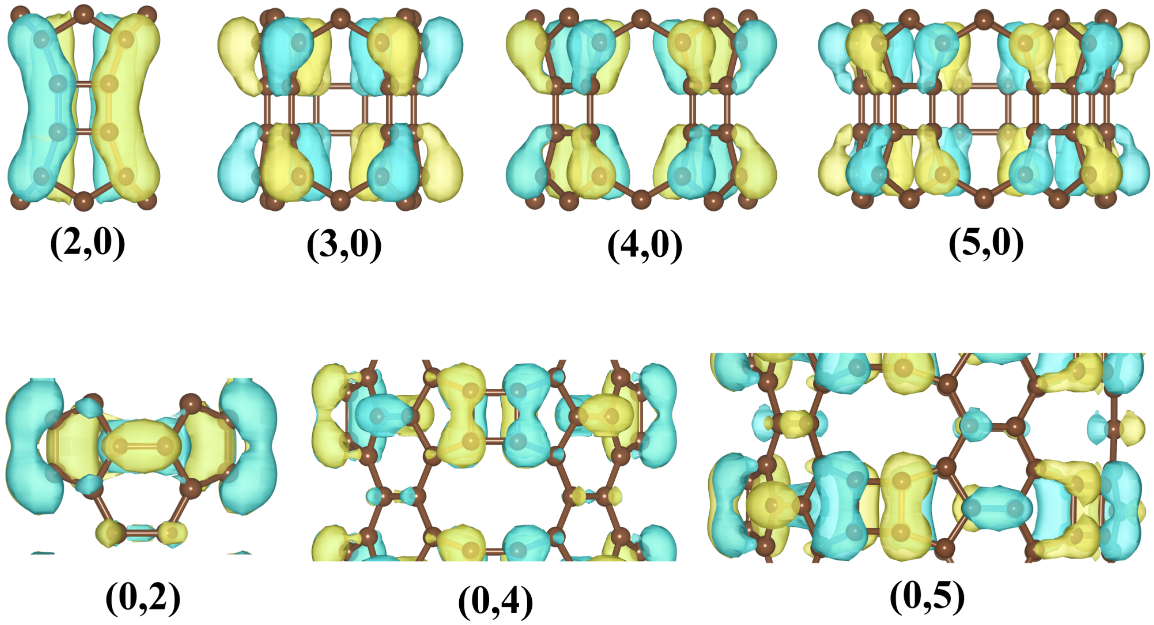

Fig. S12. Molecular orbitals for zigzag TPDH-NTs.
